# Assessing biosynthetic potential of agricultural groundwater through metagenomic sequencing: A diverse anammox community dominates nitrate-rich groundwater

**DOI:** 10.1371/journal.pone.0174930

**Published:** 2017-04-06

**Authors:** William B. Ludington, Thaddeus D. Seher, Olin Applegate, Xunde Li, Joseph I. Kliegman, Charles Langelier, Edward R. Atwill, Thomas Harter, Joseph L. DeRisi

**Affiliations:** 1 Molecular Cell Biology Department, University of California, Berkeley, United States of America; 2 Department of Land, Air and Water Resources, University of California, Davis, Davis, United States of America; 3 Department of Population Health and Reproduction, University of California, Davis, Davis, United States of America; 4 Western Institute for Food Safety and Security, University of California, Davis, Davis, United States of America; 5 Department of Biophysics & Biochemistry, University of California, San Francisco, San Francisco, United States of America; 6 Howard Hughes Medical Institute, Chevy Chase, Maryland, United States of America; National Renewable Energy Laboratory, UNITED STATES

## Abstract

**Background:**

Climate change produces extremes in both temperature and precipitation causing increased drought severity and increased reliance on groundwater resources. Agricultural practices, which rely on groundwater, are sensitive to but also sources of contaminants, including nitrate. How agricultural contamination drives groundwater geochemistry through microbial metabolism is poorly understood.

**Methods:**

On an active cow dairy in the Central Valley of California, we sampled groundwater from three wells at depths of 4.3 m (two wells) and 100 m (one well) below ground surface (bgs) as well as an effluent surface water lagoon that fertilizes surrounding corn fields. We analyzed the samples for concentrations of solutes, heavy metals, and USDA pathogenic bacteria of the *Escherichia coli* and *Enterococcus* groups as part of a long term groundwater monitoring study. Whole metagenome shotgun sequencing and assembly revealed taxonomic composition and metabolic potential of the community.

**Results:**

Elevated nitrate and dissolved organic carbon occurred at 4.3m but not at 100m bgs. Metagenomics confirmed chemical observations and revealed several Planctomycete genomes, including a new Brocadiaceae lineage and a likely Planctomycetes OM190, as well novel diversity and high abundance of nano-prokaryotes from the Candidate Phyla Radiation (CPR), the Diapherotrites, Parvarchaeota, Aenigmarchaeota, Nanoarchaeota, Nanohaloarchaea (DPANN) and the Thaumarchaeota, Aigarchaeota, Crenarchaeota, Korarchaeota (TACK) superphyla. Pathway analysis suggests community interactions based on complimentary primary metabolic pathways and abundant secondary metabolite operons encoding antimicrobials and quorum sensing systems.

**Conclusions:**

The metagenomes show strong resemblance to activated sludge communities from a nitrogen removal reactor at a wastewater treatment plant, suggesting that natural bioremediation occurs through microbial metabolism. Elevated nitrate and rich secondary metabolite biosynthetic capacity suggest incomplete remediation and the potential for novel pharmacologically active compounds.

## Introduction

The rising prevalence of drought conditions in California and elsewhere has dramatically increased demands on groundwater for irrigation and human consumption [1]. Increased reliance on groundwater resources makes evaluation of new potential sources a priority. Organic and inorganic contaminants in the water supply are prevalent in many human impacted sites such as agricultural, industrial, and municipal. However, simply measuring known sources of contamination has the potential to miss the complex effects of microbial communities in the soil and groundwater. Diverse microbial communities in subsurface environments including groundwater systems exhibit extraordinary phylogenetic diversity and metabolic complexity that has only recently become apparent using culture-independent sequencing-based analytics [2–7]. The impact of changes in water chemistry on these aquifer microbial communities, and ultimately on groundwater quality, is unknown.

Nitrogen as ammonia and nitrate are among the most ubiquitous groundwater contaminants due to widespread use in agriculture as fertilizers, as unintentional discharge in septage and effluent [8]. While crops absorb much of the applied fertilizers, significant amounts leach to groundwater. In certain regions of California’s Central Valley, over 40% of drinking water aquifers have elevated levels of nitrates [9]. The impact of these nitrogen compounds on environmental and groundwater microbial communities is not well understood, including the secondary effects on human, livestock, and wildlife health, and the potential for naturally occurring microbial populations to mineralize ammonia and nitrate to non-toxic forms. There thus exists an urgent need to understand these processes and how they may interact with remediation strategies to protect the quality of groundwater supplies.

To explore these important issues, we sampled groundwater from three adjacent wells completed at different depths that are part of a long term study on agricultural groundwater. The wells are affected to different degrees by manure, a common source of aqueous agricultural contamination. We subjected these samples to chemical analytics as well as next-generation sequencing, assembly, and genomic analysis. Our genomic analysis revealed a highly diverse microbial community dominated by many new lineages of the Candidate Phyla Radiation (CPR) and the Diapherotrites, Parvarchaeota, Aenigmarchaeota, Nanoarchaeota, Nanohaloarchaea (DPANN) superphyla and new lineages of the Planctomycete phylum with metabolic potential for both bioremediation of the contamination as well as production of potentially hazardous secondary metabolites.

## Results

### Water samples

**Fig 1:**
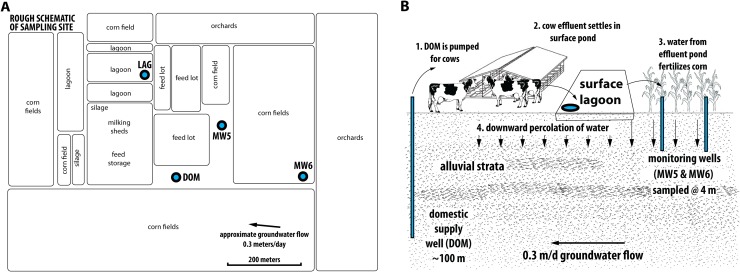
Dairy schematic. Cartoon of the sampling sites. (A) Roughly to scale layout of the sampling sites along with land use practices. (B) Illustration of vertical distribution of sampling sites. Wells, depths, and approximate characteristics of the aquifer are depicted.

We collected individual samples from each of four sites (Fig 1), the contamination source water as well as three wells. The domestic well water sample (DOM) was clear and colorless in appearance with no odor. This water is pumped from ~100 m depth and used for human and cow consumption. Cow waste is pumped into the effluent lagoon (LAG), which was cloudy and brown in appearance with an apparent odor of ammonia and feces. After settlement of particulates, the lagoon water is used as a fertilizer source for the surrounding corn fields. Monitoring well 5 (MW5) and monitoring well 6 (MW6) are located immediately downgradient and upgradient respectively from a corn field receiving lagoon water (Fig 1). These wells are screened from 3 m to 10 m below ground surface (bgs). Monitoring well samples were clear and yellowish-green in appearance with a slight organic odor. Depth to the water table for the monitoring wells was 3.4 m bgs, and the wells were sampled at a depth of 4.3 m bgs. A previous hydrologic analysis indicated that MW5 is primarily recharged from the manured corn field, MW6 receives partial recharge from the manured corn field and partial recharge from an adjacent unmanured orchard, and DOM is primarily recharged from the adjacent orchard with slight impact from the manured field [10–12].

### Chemical analysis shows high nitrate levels in shallow groundwater

While nitrate was our target analyte, many ions and nutrients in water influence the quality for drinking water and the suitability for microbial growth. Therefore, we performed a comprehensive analysis of the four samples using both standard chemical ion detection assays for ions and inductively coupled plasma mass spectrometry (ICP-MS) for trace metal detection.

For each of the 4 samples, 500 mL of raw water was drawn through a 0.45 μm filter, frozen, and sent to the UC Davis Analytical Laboratory [13] for chemical analysis of pH, dissolved organic carbon, K^+^, SO_4_^-^, NH_4_^+^, NO_3_^-^, electrical conductivity (EC), sodium adsorption ratio (SAR), Ca^++^, Mg^++^, Na^+^, Cl^-^, B, HCO_3_^-^, CO_3_^—^, Zn, Cu, Mn, Fe, Cd, Cr, Pb, and Ni (Table 1) For a point of comparison, we subjected a sample of San Francisco, CA city tap water (SF) to the same analysis.

**Table 1:** Water ion analysis. Concentration of soluble metals, ions, nitrogen, sulfur, and organic compounds in the water samples (parts per million).

Sample	DOM	LAG	MW5	MW6	SF city
sample depth	100 m	surface	4 m	4 m	tap
pH	8.17	8.16	8.65	8.63	8.09
electrical conductivity (dS/m)	0.32	5.65	2.04	0.74	0.07
sodium absorption ratio	1.80	5.50	1.60	0.70	0.70
dissolved organic carbon (DOC)	0.70	127.30	19.60	4.00	2.00
Bicarbonate (HCO_3_†)	1.90	46.60	4.90	3.10	0.30
CO_3_†	0.10	< 0.10	1.70	1.00	< 0.10
sulfate-S (SO_4_-S*)	2.30	18.00	68.80	15.30	1.10
NH_4_-N	< 0.05	332.80	< 0.05	< 0.05	0.38
NO_3_-N	4.30	0.15	105.70	21.54	< 0.05
K*	4.64	632.40	11.80	1.62	0.38
Ca*†	0.98	1.49	4.56	3.93	0.23
Mg*†	0.61	7.79	13.04	2.96	0.07
Na*†	1.58	11.96	4.74	1.24	0.27
Cl†	0.43	5.94	2.88	0.72	0.13
B*	0.13	0.60	0.43	0.15	0.03
Cr*	< 0.005	< 0.005	< 0.005	< 0.005	< 0.005
Mn*	0.073	0.010	0.530	0.117	< 0.005
Fe*	< 0.010	0.718	< 0.010	< 0.010	< 0.010
Ni*	< 0.005	0.009	< 0.005	< 0.005	< 0.005
Cu*	< 0.010	0.251	0.016	< 0.010	0.092
Zn*	< 0.005	0.033	< 0.005	< 0.005	< 0.005
Cd*	< 0.005	< 0.005	< 0.005	< 0.005	< 0.005
Pb*	0.012	0.017	0.012	0.012	< 0.010

‘<’ = below the detectable limit indicated.

* indicates ‘soluble’.

^†^ indicates ‘equivalent parts per million’.

Statistics available in S14 Table.

The pH of all the samples was similar: 8.09 (SF), 8.16 (LAG), 8.17 (DOM), 8.63 (MW6), and 8.65 (MW5). Electrical conductivity was highest in the LAG (5.65 dS/m) and lower in the other samples (MW5: 2.04 dS/m; MW6: 0.74 dS/m; DOM: 0.32 dS/m; SF: 0.07 dS/m). The SAR was also highest in the LAG (5.5) and lower in the other samples (MW5: 1.6; MW6: 0.7; DOM: 1.8; SF: 0.7), which is expected because SAR is typically correlated with the electrical conductance [14].

High levels of ammonium and nitrate occurred in the samples, with ammonium dominating the LAG sample and nitrate dominating the three well samples (Table 1). This pattern is consistent with ammonium conversion into nitrate through the action of nitrogen oxidizing microbes in the soil and aquifer [15]. Significant potassium and sulfate was also present in the samples, with the highest levels in the lagoon with lower levels in the monitoring wells and still lower levels in the deep groundwater, suggesting these ions are introduced by the LAG effluent and diluted in the groundwater.

Each sample was additionally analyzed by ICP-MS to determine the abundances of the following trace metals: B, Br, Li, Be, Na, Mg, Al, K, Ca, V, Cr, Mn, Fe, Co, Ni, Cu, Zn, Ga, As, Se, Rb, Sr, Ag, Cd, Cs, Ba, Ti, Pb, and U (Table 2). Levels of trace metals were typically highest in LAG, lower in MW5 and MW6, and lower still in DOM, suggesting the lagoon water as a source of the metals. However, the MW5 sample had high levels of arsenic, barium, manganese, strontium, selenium, and uranium, suggesting an alternate source for these trace elements. Recent reports implicated groundwater depletion as a causative factor in mobilizing some of these elements in California groundwater [16, 17]. High arsenic was also found in DOM likely due to natural occurrence in aquifer sediments. High rubidium was found in the lagoon but not in other sites. High aluminum was found in MW6 but not in other sites. We do not speculate as to the sources of the arsenic and aluminum. They are naturally occurring. Rubidium has been widely documented in cow milk [18], however the levels in milk are much lower than we detected in the lagoon. Furthermore, while almost all components of the lagoon were detected at lower levels in the wells, rubidium was not. The source of rubidium in the lagoon remains unknown [19].

**Table 2:** Water metals analysis. Metal composition of water samples by ICP-MS (parts per billion). Statistics available in S15 Table.

Metal	DOM	LAG	MW5	MW6	SF city
Na	31251.6	228151.6	89631.6	26121.6	6021.6
Mg	7340.8	80433.8	127563.8	35233.8	867.8
K	4239.1	592987.1	10517.1	1473.1	401.5
Ca	19545.1	37565.1	117055.1	85865.1	4719.1
*total*	62376.6	939137.6	344767.6	148693.6	12010.0
B	113.6	552.8	353.4	126.6	25.3
Br	51.8	326.5	216.4	84.6	0.0
Li	5.1	12.0	8.4	7.8	1.1
Be	0.0	0.0	0.0	0.0	0.0
Al	0.0	0.0	0.0	209.2	2.9
V	26.8	2.1	37.4	30.6	0.2
Cr	0.0	1.0	0.1	0.3	0.0
Mn	65.4	8.5	488.3	114.2	0.7
Fe	0.0	663.9	13.4	15.1	1.2
Co	0.0	15.8	3.2	0.5	0.0
Ni	0.0	9.9	4.5	2.8	0.1
Cu	0.5	235.2	26.6	3.5	69.8
Zn	8.8	32.6	7.1	8.4	8.9
Ga	0.0	0.0	0.2	0.1	0.0
As	15.4	4.9	9.9	3.2	0.3
Se	0.0	0.0	5.6	0.0	0.0
Rb	1.1	459.0	0.6	0.7	0.6
Sr	222.3	321.4	2386.0	867.6	33.9
Ag	0.0	0.0	0.0	0.0	0.0
Cd	0.0	0.0	0.0	0.0	0.0
Cs	0.0	1.2	0.1	0.2	0.2
Ba	82.2	21.7	469.7	64.6	8.2
Tl	0.0	0.3	0.1	0.0	0.0
Pb	0.0	0.4	0.1	0.0	0.0
U	1.6	0.2	54.8	9.0	0.2
*total*	594.7	2669.5	4085.9	1548.9	153.7

### Pathogenic microbes cultured from lagoon but not groundwater

Each water sample was tested for the presence of USDA pathogenic bacteria by inoculating liquid enrichment media and plating on selective nutrient media [20–22]. The specific pathogens tested for were *Salmonella*, *Enterococcus*, *Escherichia coli*, and *E*. *coli* O157. Of these, *Enterococcus*, *Escherichia coli*, and *E*. *coli* O157 were detected in the LAG sample, but no pathogens were detected in any of the groundwater samples. Previous samplings from these and other similar monitoring wells on dairies did reveal the presence of USDA pathogens [21]. However, it is not known how long these pathogens remain viable in the groundwater, and lagoon water had not recently been applied to the field where the monitoring wells are located. Our failure to detect these pathogens in groundwater suggests that they have a limited residence time.

### Taxonomic composition of the microbial communities

We asked whether the microbial composition of the water samples matched the chemical and culture-based observations. We analyzed the water microbial communities for DOM, LAG, MW5, and MW6 by constructing a whole metagenome library for each water sample and shotgun sequencing to a depth of ~50 million paired end 101 bp reads.

We analyzed taxonomic makeup of the samples both by 16S rRNA gene profiling and whole metagenome assembly. First, we used EMIRGE [23] to do reference-guided assembly of 16S ribosomal subunit genes and abundance estimation for each of our shotgun sequencing libraries [23]. We then assigned taxonomy to the 16S assemblies using the RDP web interface [24] (Table 3). Second, we assembled all of our reads and binned genomes from the assembled contigs and then assigned taxonomy to the genomic bins using RAPSEARCH [25] to the UniProt UniRef100 database [26] (S1–S4 Tables).

**Table 3:** Taxonomic relative abundances for the four water samples based on 16S rRNA gene abundances. Frequencies of taxa present at > 1.1% relative abundance are displayed. Standard error of the proportion is less than 0.4% for all observations. Color scaling relative abundances: red = high; yellow = moderate; green = low.

Taxonomy: Kingdom_Phylum_Genus	LAG	DOM	MW5	MW6
Archaea_Crenarchaeota_Thermocladium			22.9%	28.1%
Archaea_Woesearchaeota_Woesearchaeota AR16		1.09%	12.2%	5.38%
Bacteria_Chloroflexi_Dehalogenimonas			11.0%	
Bacteria_Planctomycetes_Candidatus Brocadia sp.		1.53%	10.9%	11.0%
Bacteria_Microgenomates_Microgenomates_genera			9.42%	2.68%
Bacteria_Parcubacteria_Parcubacteria_genera	1.23%		7.66%	1.25%
Plantae_Cyanobacteria/Chlorella_Chlorella			7.37%	5.40%
Archaea_Pacearchaeota_Pacearchaeota AR13			3.01%	
Bacteria_Firmicutes_Thermacetogenium			2.85%	
Bacteria_Verrucomicrobia_Opitutus			2.46%	5.77%
Bacteria_Omnitrophica_Omnitrophica_genera			2.35%	
Bacteria_Planctomycetes_Aquisphaera			2.15%	1.61%
Archaea_Thaumarchaeota_Nitrosopumilus			2.10%	1.87%
Bacteria_Chloroflexi_Bellilinea			1.89%	
Bacteria_Acidobacteria_Acanthopleuribacter				13.0%
Archaea_Euryarchaeota_Methanomassiliicoccus				6.98%
Bacteria_Spirochaetes_Leptonema				2.25%
Bacteria_Chloroflexi_Levilinea	1.94%			1.85%
Bacteria_Acidobacteria_Candidatus Koribacter				1.72%
Bacteria_Bacteroidetes_Flavitalea				1.70%
Bacteria_Firmicutes_Thermanaeromonas				1.46%
Bacteria_∂-Proteobacteria_Haliangium				1.25%
Bacteria_Bacteroidetes_Sediminibacterium				1.16%
Bacteria_Firmicutes_Domibacillus		37.1%		
Bacteria_Proteobacteria_Sphingomonas		11.1%		
Bacteria_Nitrospirae_Nitrospira		5.85%		
Bacteria_Proteobacteria_Syntrophorhabdus	1.54%	5.24%		
Bacteria_Proteobacteria_Aquabacterium		3.88%		
Bacteria_Proteobacteria_Methylobacterium		3.54%		
Bacteria_Bacteroidetes_Rikenella	21.9%	2.87%		
Bacteria_Proteobacteria_Acidovorax		2.49%		
Bacteria_Proteobacteria_Propionivibrio		2.08%		
Bacteria_Actinobacteria_Ornithinimicrobium		1.65%		
Bacteria_Firmicutes_Desulfovirgula		1.52%		
Bacteria_Nitrospirae_Leptospirillum		1.50%		
Bacteria_Proteobacteria_Thiolamprovum	3.62%	1.25%		
Bacteria_Bacteroidetes_Anaerorhabdus	8.04%	1.19%		
Bacteria_Proteobacteria_Halochromatium	8.20%	1.18%		
Bacteria_Tenericutes_Acholeplasma	7.61%	1.16%		
Bacteria_Proteobacteria_Oxalicibacterium		1.13%		
Bacteria_Tenericutes_Asteroleplasma	4.67%			
Bacteria_Synergistetes_Cloacibacillus	3.36%			
Bacteria_Firmicutes_Thermotalea	3.00%			
Bacteria_Cloacimonetes_Candidatus Cloacamonas	2.76%			
Bacteria_Firmicutes_Anaerovorax	2.71%			
Bacteria_Firmicutes_Proteiniclasticum	2.48%			
Bacteria_Bacteroidetes_Prolixibacter	2.39%			
Bacteria_Firmicutes_Syntrophothermus	1.54%			
Bacteria_Firmicutes_Turicibacter	1.51%			
Bacteria_Bacteroidetes_Arcticibacter	1.32%			
Bacteria_Verrucomicrobia_Subdivision3_genera	1.27%			
Bacteria_Spirochaetes_Sphaerochaeta	1.24%			
Bacteria_Verrucomicrobia_Subdivision5_genera	1.18%			
Bacteria_Bacteroidetes_Leadbetterella	1.13%			
total % displayed	85%	87%	98%	94%
**number of genera represented (54 total):**	**21**	**19**	**14**	**18**
total number of 16S reads	30,244	29,440	9,654	14,934

The two metagenomic approaches we took are in good agreement with each other and with the water chemistry, however, we did not detect any of the cultured pathogens from the surface water by sequencing, suggesting they are rare. The shallow groundwater communities of MW5 and MW6 have similar species composition and are similar to the activated sludge bioreactor communities recently reported by Speth *et al* [27] (S5 Table), a community sampled from the nitrogen removal stage of sewage wastewater treatment. However, in addition to observing 10 of the 12 phylogenetic groups reported by Speth, we additionally see 13 more in the groundwater. Specifically enriched are prokaryotes from the recently described nano-bacterial Parcubacteria (OD1) and Microgenomates (OP11), nano-archaeal DPANN and Thaumarchaeota-Aigarchaeota-Crenarchaeota-Korarchaeota (TACK) superphyla [2, 3] as well as two distinct clades of Planctomycetes: the OM190 group, and the anammox Brocadiaceae group.

Examining the EMIRGE data at abundances over 5%, the Archaea dominate, with the Crenarchaeote, *Thermocladium* (23% and 28% abundance in MW5 and MW6 repsectively), Woesearchaeota (12% and 5.4% respectively), and *Methanomassiliicoccus* (0% and 7.0% respectively). The Bacteria include anammox Planctomycetes from the *Brocadia* group (11% in both MW5 and MW6), *Acanthopleuribacter* (0% and 13% respectively), Microgenomates genera (9.4% and 2.7% respectively), *Dehalogenimonas* (11% and 0% respectively), Parcubacteria genera (7.7% and 1.3% respectively), and *Opitutus* (2.5% and 5.8% respectively). The other major lineages in these samples include many known as nitrifiers, denitrifiers and methylotrophs as well as the heterotrophic eukaryote, *Chlorella* (Plantae), at 7.4% and 5.4% respectively.

In contrast to the similarities seen between the two shallow groundwater samples, the DOM and LAG samples each have their own distinct communities. The DOM sample is dominated by *Domibacillus* (37%) followed by *Sphingomonas* (11%) and *Nitrospira* (5.9%). Anammox genomes are more rare (~2% abundance) in the deep groundwater, matching the trend seen in nitrate concentrations. The surface water (LAG) is dominated by *Rikenella* (22%), which is known from animal feces. Several other likely animal-associated genera are abundant, including *Anaerorhabdus* (8.0%) and *Acholeplasma* (7.6%), as well as a photosynthetic bacterium, *Halochromatium* (8.2%). Overall, the taxonomic representation in the water samples matches well with expectations based on the chemical data.

We note that several taxa appear unexpectedly in both the DOM and LAG samples, and we suspect these are contaminants in DOM from airborne dust. Specifically, *Rikenella*, the most dominant member of LAG, is present at 2.9% abundance in DOM. Likewise *Anaerorhabdus*, *Coprobacillus*, *Halochromatium*, and *Acholeplasma* are abundant at >5% in LAG and ~1% in DOM. Airborne dust was ever-present while we sampled, and despite extensive containment efforts, some exposure of the apparatus to dust occurred [20]. However, the rest of the DOM metagenome is remarkably distinct from the other samples, suggesting that, with the exception of the known contaminants, it is still representative of the deep groundwater. We eliminated all suspected contaminant genomes from further analysis.

### Whole metagenome assembly and analysis

We assembled genomic bins (i.e. high-coverage but incomplete genomes) using the following pipeline: **(1)** IDBA_UD [28] initial assembly, **(2)** REAPR [29] breaking of misassemblies, **(3)** VizBin [30] binning of contigs into draft genomes using 5-mer frequency and coverage information for visual aid, **(4)** additional manual bin cleanup based on contig coverage distribution (as assessed by Bowtie2 [31]), **(5)** taxonomic assignment of bins based on RAPSEARCH [25] to the UniProt UniRef100 database [26]. Selected bins were assembled further **(6)** by PRICE [32] targeted assembly and REAPR correction of misassemblies. **(7)** Genome quality was assessed throughout using CheckM [33]. Further details are in the methods.

Overall we assembled and refined 79 unique genomic bins from the three groundwater samples (Table 4). An additional 51 bins were made from the LAG sample (S4 Table) and were used to determine probable contaminants in other samples, but no refinement of these bins was attempted, as we were interested in the properties of the groundwater communities. Contamination was only detected for the DOM sample as previously discussed. No evidence of overlap with the surface water was seen in either MW5 or MW6.

**Table 4:** Summary of genomic bins.

bin ID	top taxonomic call	median coverage (RPKM)	total bin size (bp)	%GC	longest contig (bp)	# of contigs	N50	single copy marker genes (of 111)	total # related genome bins	# related bins by sample
MW5-40_1	CPR (OD1) Parcubacteria	27	738,670	51%	51,322	42	22,985	87	15	MW5 (10), MW6 (3), DOM (2)
MW5-01_1	DPANN (Woesearchaeota)	27	1,647,473	46%	85,567	94	22,298	34	11	MW5 (8), MW6 (1), DOM (2)
MW5-33_1	CPR (OP11) Microgenomates	15	1,127,108	41%	423,501	10	349,088	86	10	MW5 (6), MW6 (3), DOM (1)
MW6-03	Planctomycetes (Brocadiaceae)	3	2,230,970	52%	51,877	208	14,077	99	7	MW5 (2), MW6 (3), DOM (2)
MW5-32_1	other CPR	5	893,697	46%	59,583	51	25,307	95	5	MW5 (3), MW6 (2)
MW5-13_1	OP3 (Omnitrophica)	5	2,621,710	42%	95,713	151	24,451	100	4	MW5 (1), DOM (3)
MW5-17_1	NC10 (Methylomirabilis)	6	2,733,247	59%	128,287	107	37,739	103	3	MW5 (1), DOM (2)
MW6-01	Nitrospirae	11	2,561,774	46%	131,194	161	23,038	94	3	MW6 (1), DOM (2)
MW5-12_2	other DPANN	5	880,039	45%	48,260	58	19,549	26	2	MW5 (1), DOM (1)
MW6-18	Chloroflexi	2	1,662,667	50%	29,959	255	7,912	87	2	MW5 (1), MW6 (1)
MW6-04	Chlorobi	5	3,770,623	45%	198,151	106	56,504	106	2	MW6 (2)
MW6-08	Nitrospinae/Tectomicrobia	4	7,190,427	58%	123,483	868	10,766	97	2	MW6 (2)
DOM-09	Bacteroidetes (Bacteroidales)	5	2,062,333	49%	253,464	38	152,399	100	2	DOM (2)
MW5-24_1	Cyanobacteria	4	518,458	53%	45,971	60	9,946	0	1	MW5 (1)
MW6-06	Spirochaete	4	5,712,367	59%	94,173	413	20,386	98	1	MW6 (1)
MW6-07	Acidobacteria	11	7,709,548	34%	348,446	315	69,143	101	1	MW6 (1)
MW6-09	Planctomycetes (likely OM190)	10	8,304,162	62%	120,147	381	47,364	102	1	MW6 (1)
MW6-12	TACK	4	1,651,017	40%	75,874	93	27,817	31	1	MW6 (1)
DOM-01	Firmicutes (Bacilli)	21	4,020,846	46%	81,760	214	27,212	94	1	DOM (1)
DOM-05	γ-Proteobacteria	3	1,942,763	64%	43,739	276	7,042	82	1	MW6 (1)
DOM-13	bacteriophage	4	105,149	29%	29,834	9	14,045	0	1	DOM (1)
DOM-14	Bacteroidetes (Flavobacteriia)	3	913,095	33%	20,267	135	6,773	49	1	DOM (1)
DOM-20	α-Proteobacteria	8	4,639,849	66%	182,742	202	38,128	103	1	DOM (1)
DOM-23	β-Proteobacteria	3	2,792,956	63%	39,022	397	6,827	60	1	DOM (1)
								**total:**	**79**	

The most abundant taxa in the partial genomic bins were from the CPR Parcubacteria (OD1) (n = 15 bins), followed by the DPANN Woesearchaeota (n = 11 bins), and the CPR Microgenomates (OP11) (n = 10 bins). While the OD1 and OP11 lineages have previously been found in association with anammox communities [27]. The high relative abundance and diversity of CPR and DPANN genomes at over 50% of the community is notably higher than previous reports (Table 4).

The next highest abundance of our genomic bins were from the Planctomycete Brocadiaceae family (n = 7).

We made genomic bins of many of the other key members of the wastewater anammox community [27], including Omnitrophica (OP3) (4 bins), Nitrospirae (3 bins), Chloroflexi (2 bins), Chlorobi (2 bins), Bacteroidales (2 bins), Acidobacteria (1 multistrain bin), and γ-Proteobacteria (1 bin) (Tables 4 and 5). Additionally, we assembled partial genomes from the Nitrospinae/Tectomicrobia group (2 bins), Spirochaete (1 bin), a Planctomycete from the OM190 family, an archaeon from the TACK radiation, a Firmicute (*Domibacillus*) that was dominant in the DOM well, a Bacteroidetes (Flavobacteriia), an α-Proteobacterium, and a β-Proteobacterium. Finally, we assembled numerous phage bins. Only one is included here (DOM-13; S10 Table), but evidence of bacteriophage was abundant.

**Table 5:** Summary of KEGG pathway representation by phylogenetic grouping. See S13 Table also.

taxon	nitrogen metabolism	sulfur metabolism	nucleotide synthesis	flagellar assembly	chemotaxis	terpenoid synthesis	ATPase	secretion systems	co-F420 (methane)	B12
**DPANN**	sparse coverage	sparse coverage	high coverage	sparse individual coverage; complete as a group	moderate coverage	either mevalonate or MEP/DOXP	A-type ATPase	secSRP	one MW6 genome has pathway	users not producers
**OP11**	sparse coverage	assimilitory sulfate reduction	high coverage	sparse individual coverage; complete as a group	moderate coverage	mevalonate	F-type ATPase	secSRP	not present	one genome does partial synthesis; others none
**OD1**	sparse coverage	sparse coverage	high coverage	sparse individual coverage; near-complete as a group	moderate coverage	not present	F-type ATPase	secSRP	one MW5 genome has pathway	not present
**Methylomirabilis**	high coverage	high coverage	high coverage	sparse coverage	sparse coverage	MEP/DOXP	F-type ATPase	II, secSRP, Tat	high coverage	sparse coverage
**Omnitrophica**	high coverage	high coverage	high coverage	sparse coverage	sparse coverage	MEP/DOXP	F-type ATPase	II, secSRP, Tat	not present	full producer
**Nitrospira**	high coverage	high coverage	high coverage	high coverage	high coverage	MEP/DOXP	A-type & F-type	I, II, SecSRP, Tat	not present	full producer
**Brocadiaceae**	high coverage	high coverage	high coverage	high coverage	high coverage	MEP/DOXP	A-type & F-type	II, IV (1), VI (1), SecSRP, Tat	not present	full producer
**Chlorobi**	moderate coverage	moderate coverage	high coverage	high coverage	moderate coverage	both mevalonate and MEP/DOXP	F-type ATPase	II, IV, VI, SecSRP, Tat,	moderate coverage	partial synthesis
**Bacteroidales**	sparse coverage	sparse coverage	high coverage	sparse coverage	moderate coverage	MEP/DOXP	A-type & F-type	SecSRP, Tat	not present	partial synthesis
**Nitrospinae**	high coverage	high coverage	high coverage	high coverage	high coverage	MEP/DOXP	F-type ATPase	I, II, IV, VI, SecSRP, Tat	high coverage	full producer

### Many novel Planctomycete genomes

**Fig 2:**
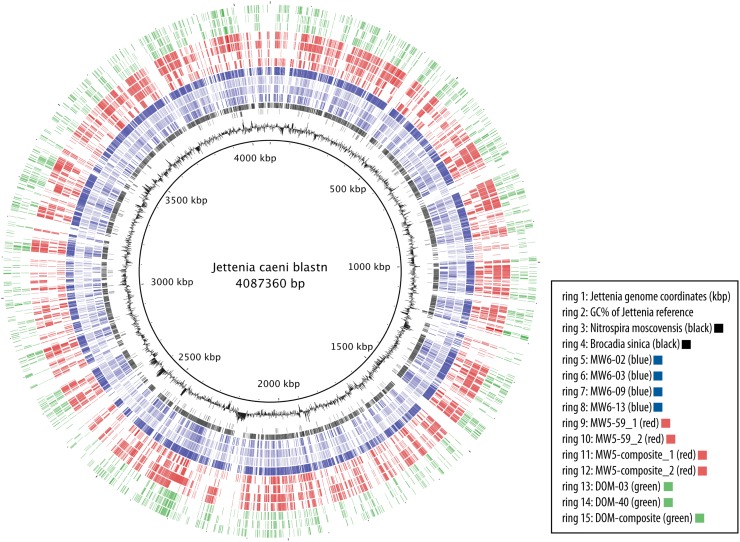
Homology of assembled Brocadiaceae genomes to the *Jettenia caeni* reference genome. BRIG [37] was used to compare the Brocadiaceae genomic bins to the reference *Jettenia caeni* by BLASTN [38]. Radial colored bars in the concentric rings indicate nucleotide homology (30–100%). See graphical legend for ring identities. MW5 bins are shown in green, MW6 in red, and DOM in blue. Contig order is that of the reference genome.

While many potentially interesting and novel genomes were isolated from this community, we focus here on the Brocadiaceae Planctomycetes, which oxidize ammonium under anaerobic conditions in a specialized organelle called the anammoxosome that protects the cells from the toxic hydrazine intermediate products of the biochemical reaction [34–36]. Anammox genomes are highly prevalent in the MW5 and MW6 samples (11% abundance each) and also occur in the DOM sample (2%). We were able to make several near-complete assemblies of these genomes (as measured by single copy gene abundance; S1–S4 Tables), however, the genome size of ≈2 Mb for many of these genomes (e.g. MW5-59_1 and MW5-59_2) was well below the ≈4 Mb seen in other members of this family [27, 35]. The coverage of some of these small genomes is rather high (e.g. 48 RPKM for MW5-59_2; S1 Table), making it seem plausible that the true genome size is reduced. In an attempt to resolve this discrepancy, we rebinned the MW5 anammox genomes using less stringent criteria, and found increased but still incomplete coverage of the Brocadiaceae reference genomes (‘MW5-composite’ in Fig 2, S3 Fig). These composite genomes were multi-strain chimeras, as indicated by conserved single copy gene occurrences increasing above one (using CheckM see Methods). The fact that merging multiple strains of the same species did not give complete coverage of the single copy genes is in agreement with the hypothesis that the true genome size is small but further sampling would be needed to confirm the hypothesis. In any event, we have not been able to resolve the discrepancy in genome size in the present study.

**Fig 3:**
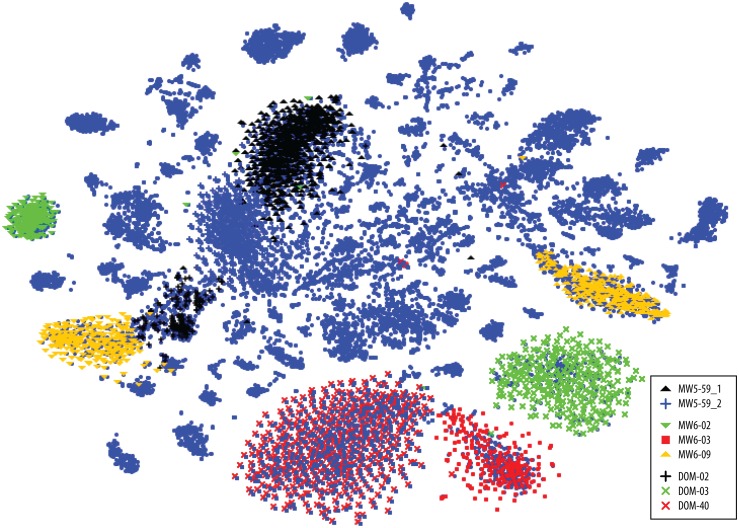
VizBin-based clustering of contigs based on the pentanucleotide (5-mer) frequency distribution shows distinct anammox genomes. Each dot indicates a specific contig 1000 to 5000 bp in length. Color code in the key indicates identity of the dots by shape and color.

**Fig 4:**
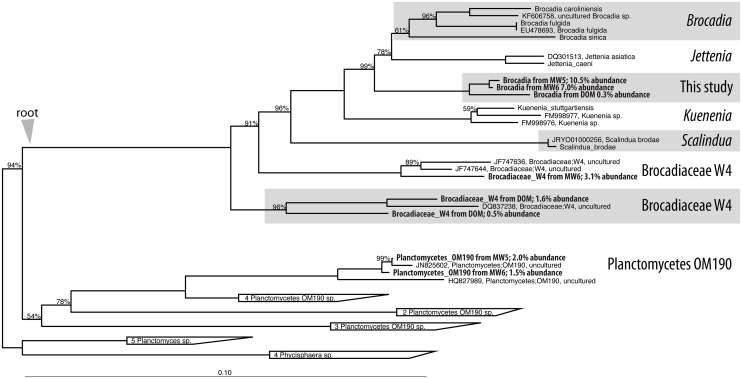
16S phylogeny of the Planctomycetes on shows diversity within two distinct groups, the Brocadiaceae and the OM190 clade. Bold samples indicate they are from this study. Scale bar indicates 10% sequence divergence.

The phylogenetic placement is apparent by homology with the *Brocadia sinica* and *Jettenia caeni* reference genomes (Fig 2). The separation of genomic bins shown by pentanucleotide clustering (Fig 3) suggests multiple *Brocadia*-like genomes coexist in MW5, MW6 and DOM. A 16S phylogeny supports this observation (Fig 4). We refer to these genomic bins herein as MW5-59_1, MW5-59_2, MW6-02, MW6-03, MW6-13, DOM-02, DOM-03, and DOM-40 (see S1–S3 Tables for additional genomic bin metrics).

We also assembled an 8.3 Mb Planctomycete genome with similarity to the Planctomycetaceae family within the Planctomycetes (MW6-09). The larger genome size indicates the genome is not of the Brocadiaceae, which have genome sizes around 4 Mb. Whole genome sequence comparison of MW6-09 to the available reference Planctomycetes showed highest similarity to *Singulisphaera acidiphilus* (S1 Fig), however, the similarity even to *Singulisphaera* was not especially high, indicating that this genome is truly diverged from the reference genomes. Examining the 16S alignment suggests the genome could be from the OM190 group of Planctomycetaceae (Fig 4), a group with no sequenced genomes (to our knowledge). We caution, however, that while we could link the EMIRGE-assembled OM190 16S gene with the MW6-09 genome using targeted assembly (PRICE), multiple 16S fragments could be linked to the genome, thus our placement MW6-09 as an OM190 Planctomycetaceae should be revisited when new, related genomes are discovered.

To determine whether the anammox strains were unique to their respective bins or overlapping, we used VizBin to perform additional kmer distribution-based clustering of all the Planctomycete contigs together. Six distinct clusters are apparent (S2 Fig), with MW5-59_1, MW5-59_2, and MW6-13 overlapping. We next refined the genomic bins by combining the anammox genomes from MW5, MW6, and DOM and repicking chimeric bins. We then performed a single round of assembly using PRICE in order to merge the contigs. Overall, little improvement in bins was made. However, inter-strain contamination was reduced, and the DOM-02 bin was substantially improved by adding ≈20% more contigs from MW6 that co-clustered (43 new contigs were added to the initial 208).

The 16S phylogengy indicates the bins come from three distinct lineages (Fig 4). The abundant MW5 and MW6 bins come from a new lineage that is intermediate between *Jettenia* and *Kuenenia*. The abundant DOM bins and one of the MW6 bins come from two different lineages within the Brocadiaceae W4 group. As there are no sequenced members of these lineages to use as reference, we aligned our bins to the closest available reference draft genomes of *Brocadia*, *Jettenia*, *Kuenenia* and *Scalindua* species (Fig 2, S3 Fig). Reflecting the 16S phylogeny, the best homology was to *Jettenia* for the abundant MW5 and MW6 bins, and lower homology was seen for the DOM bins and MW6-02. As previous reports have noted low diversity of anammox genomes within a given sample (e.g. [39]), we find it noteworthy that as many as three distinct anammox genomes coexist within a single groundwater well. To confirm that all of these genomes were true anammox metabolizers, we checked for hydrazine conversion genes (hydrazine synthase, hydrazine oxidoreductase, and hydrazine hydrolase) by BLASTX [40]. Confirming that they are indeed anammox organisms, all Brocadiaceae genomes showed good coverage of the hydrazine database, and MW6-09, which is phylogenetically places as a non-anammox Planctomycete, did not have BLASTX hits.

### Metabolic pathways of genomic bins

We analyzed the biochemical potential of the genomic bins in two ways focusing on pathways and modules rather than on individual proteins (due to the known caveat that existing databases are prone to false positive and false negative errors at the protein level for such poorly resolved taxa). This analysis was based on taxonomic placement rather than on the well of origin. First, we mapped the contigs from each draft assembly to the database of KEGG orthologs and used KEGG Mapper (http://www.genome.jp/kegg/tool/map_pathway.html) to visualize the results. Second, we used antiSMASH [41–44] to detect potential secondary metabolite biosynthetic gene clusters. The results reveal variation between genomic bins as well as pathways for potential community interactions linking nitrogen and sulfur metabolic pathways in the groundwater.

#### KEGG pathway comparisons

To determine what functional genes are present in the water microbiota, we first aligned all of our contigs in each of the dairy water samples to the KEGG prokaryote database [45, 46] (S6 Table) and evaluated trends at the whole metagenome level. Overall, we see enrichment of phosphotransferase (PTS) systems, two-component systems, ABC transporters, and terpenoid production. The PTS systems are particularly high in the nutrient poor (a.k.a. clean) DOM sample, consistent with the idea that there is a selective pressure driving acquisition of nutrients in nutrient-poor environments. However, no clear signature of different modes of nitrogen metabolism is indicated when examining the aggregated data for each sample. Thus, we examined the individual genomic bins to get a broad understanding of their biochemical potential.

We focused on the KEGG pathways for nitrogen metabolism, sulfur metabolism, flagellar assembly, chemotaxis, ABC transporters, two-component systems, terpenoid synthesis, ATPase family, secretion systems, cofactor F420 (for methane redox), and B12 production since these pathways showed the most variability across the genomic bins. We include nucleotide synthesis as a positive control, since all of the complete bins have good coverage of the nucleotide metabolism pathways. Because many of the genomic bins were partially incomplete, we aggregated KEGG maps from related species in order to get a more coherent picture of the pathway representation as a function of phylogeny (Table 5, S13 Table).

Overall, we see sparse coverage of nitrogen metabolism by the CPR, and DPANN genomes, while Methylomirabilis, Omnitropica, Nitrospira, Brocadia, and Nitrospinae had high coverage. The Bacteroidales also had sparse coverage of nitrogen metabolism, and the Chlorobi had intermediate coverage of the pathway, indicating that not all genomes in the community are directly involved in nitrogen metabolism. The same pattern was true of sulfur metabolism, with the exception that OP11 has the module for assimilatory sulfate reduction, which is consistent with the work of Canfield [47] showing that sulfur and nitrogen redox pathways are coupled in the oxygen minimum zone of the oceans. Methane metabolism, indicated by presence of the coenzyme F420, was present in one DPANN bin, one OD1 bin, Methylomirabilis, and Nitrospinae. Intermediate coverage of this module was seen for Chlorobi, supporting the observations of Speth *et al* [27] that species have diverse and overlapping niches within the anammox community and Shen *et al* [15] that methane oxidation co-occurs with anammox.

For oxidative phosphorylation, distinct ATPases were seen between the phyla. OP11, OD1, Methylomirabilis, Omnitrophica, Chlorobi, and Nitrospinae have the F-type ATPase, while DPANN, has the A-type ATPase, and Nitrospira, Brocadiaceae, and Bacteroidales have both F-type and A-type ATPases.

In terms of acquiring nutrients from the environment, the CPR genomes were deficient in ABC transporters besides phosphate. The DPANN have slightly more, but the rest of the genomes each have significant coverage of ≈15 ABC transporters each. Coverage of two-component systems was consistent across all genomes for phosphate, while either nitrogen or nitrate was present for all except OP11. Twitching motility was indicated for OP11 as well as for OD1, and Chlorobi. High coverage of the chemotaxis pathway was seen only in the Nitrospira, Brocadiaceae, and Nitrospinae, with moderate coverage seen in the DPANN, OP11, OD1, Chlorobi, and Bacteroidales. Methylomirabilis and Omnitrophica appear to lack pathways for both chemotaxis and flagellar assembly, whereas Nitrospira, Brocadiaceae, and Nitrospinae have the complete pathways, and Chlorobi and Bacteroidales have most of the chemotaxis pathway but Chlorobi has the complete flagellar pathway and Bacteroidales lack it entirely.

In terms of biosynthetic capabilities, Omnitrophica, Nitrospira, the Brocadiaceae, and Nitrospinae have complete vitamin B12 pathways, while Chlorobi and Bacteroidales have the latter half of the pathway as does one OP11 genome, while the other OP11 genomes as well as the OD1 genomes lack B12 production entirely. Methylomirabilis has sparse coverage of the pathway, and the DPANN genomes have genes for converting B12 to the active form. These results indicate that B12 sharing is likely an active part of the anammox community metabolism.

Terpenoid synthesis showed clear segregation into the mevalonate and the MEP/DOXP (non-mevalonate) pathways. Methylomirabilis, Omnitrophica, Nitrospira, Brocadiaceae, Bacteroidales, and Nitrospinae all have only the non-mevalonate pathway, whereas Chlorobi has both pathways, and DPANN use either one pathway or the other but not both. Within the CPR, OP11 has the mevalonate pathway, and OD1 has neither pathway.

Wide variation was seen in the secretion systems, with the DPANN, OP11, and OD1 using only the SecSRP system, Bacteroidales using the SecSRP and Tat systems, Methylomirabilis and Omnitrophica using the SecSRP, Tat, and Type II systems, and Nitrospira using the SecSRP, Tat, Type I and II systems. The Brocadiaceae and Chlorobi use the SecSRP, Tat, Type II, IV and VI systems. And finally, Nitrospinae uses the the SecSRP, Tat, Type I, II, IV and VI systems.

Comparison of KEGG pathway coverages with the available reference genomes showed similar results, supporting the hypothesis of niche specialization in a shared community metabolism.

#### AntiSMASH evaluation of secondary metabolite potential

We next examined the genomic bins using antiSMASH 2.0 [42]. A rich range of secondary metabolites is predicted for the genomic bins (Table 6, S7 Table). The majority of the clusters overall were uncategorized (in the “cf_putative” category), followed by saccharides and fatty acids. non-ribosomal peptide synthases, bacteriocins, and terpenes, and polyketide synthases were also common. Arylpolyenes, lasso- and lantipeptides also were predicted as was one instance each of a siderophore and butyrolactone. MW5 had 229 clusters in 33 bins. MW6 had 371 clusters in 22 bins. DOM had 10 clusters in 158 bins. Notably, the CPR genomes that dominate the water samples have few predicted secondary metabolites on average. Because MW5 was dominated by these genomes, its density of clusters is correspondingly lower. However, some of the individual CPR bins are dense with biosynthetic clusters (up to 17 in one OP11). Thus while poor representation of CPR in existing databases may reduce utility of this approach, some of the genomes certainly have detectable clusters.

**Table 6:** Summary of antiSMASH biosynthetic gene cluster predictions by phylogenetic grouping.

taxonomy	putative	saccharide	fatty acid	NRPS	bacteriocin	terpene	polyketide synthase	butyrolactone	lantipeptide / lassopeptide	arylpolyene	siderophore	unclassified biosynthetic cluster	avg total clusters per genome	max # clusters per genome	# of related bins	predicted products
**Planctomycetes (likely OM190)**	24	5	7	31	3	1	total (18); trans AT (7); type I (10); type III (1), other	-	lasso (2)	2	-	3	77	77	1	AWQC131C; ladderane; APE_Vf; Pellasoren; anatoxin;
**Acidobacteria**	23	7	2	1	3	3	type III	-	lanti (2)	-	-	2	41	41	1	Dkxanthene; Mithramycin
**Sphingomonas**	29	8	2	-	1	1	(2) Type I, III	-	lasso (1)	-	-	-	41	41	1	capsular polysaccharide, diutan polysaccharide; Astaxanthin dideoxyglycoside
**Spirochaete**	19	6	6	2	1	-	(4) type I (2), II, III	1	-	-	-	1	36	36	1	Marinopyrrole; LPS
**Domibacillus**	17	3	3	-	2	1	(1) type III	-	-	-	1	-	27	27	1	Emulsan, Bacillomycin, Exopolysaccharide; O-antigen, S-layer glycan; Bacillomycin; Carotenoid
**Chlorobi**	9	4	3	-	-	1	-	-	lasso (1)	3	-	-	21	21	2	colanic acid; flexirubin; S-layer glycan; resorcinol; flexirubin (2); ravidomycin; azinomycin B, carotenoid
**Entotheonella**	8	4	4	1	1	1	type II	-	-	-	-	1	20	27	2	Caprazamycin; Grincamycin
**Planctomycetes (Brocadiaceae)**	4	6	4	-	1	1	-	-	-	-	-	-	15	19	6	Ladderane, LPS, O & K antigen, Vicenistatin, Exopolysaccharide, Colabomycin
**Methylomirabilis**	5	4	1	-	-	3	type III	-	-	-	-	1	14	14	1	Avilamycin_A; Saframycin_A; Heme D1
**OP3**	4	6	2	-	-	-	-	-	-	-	-	-	13	28	5	Stambomycin, Glycopeptidolipid, UK-68597, S-layer glycan
**Nitrospirae**	4	3	2	-	2	1	other	-	-	-	-	-	13	13	1	Polyhydroxyalkanoic acid
**Bacteroidales**	3	1	2	1	-	1	-	-	-	2	-	1	10	11	2	O & K antigen; Flexirubin
**Chloroflexi**	6	2	1	-	-	-	-	-	-	-	-	-	9	11	2	-
**DPANN**	2	4	1	-	-	-	-	-	-	-	-	-	7	10	10	S-layer_glycan, Proteusin, Alkaloid, Elaiophylin
**OP11**	2	5	-	-	-	-	-	-	-	-	-	-	7	17	10	S-layer_glycan, Exopolysaccharide, Lipomycin, Stambomycin
**other CPR**	3	3	-	-	-	-	-	-	-	-	-	-	6	7	2	polysaccharide; S-layer glycan
**Cyanobacteria**	-	-	-	5	-	-	-	-	-	-	-	1	6	6	1	-
**OD1**	1	3	-	-	-	-	-	-	-	-	-	-	5	7	13	colanic acid, LPS
**TACK**	4	-	-	-	-	-	-	-	-	-	-	-	4	4	1	-
**∂-Proteobacteria**	-	-	-	1	1	-	-	-	-	-	-	-	2	2	1	-
**TM7**	1	-	-	-	-	1	-	-	-	-	-	-	2	2	1	-

Grouping the genomes phylogenetically (Table 6), the most clusters occur in the Planctomycetes OM190 (77 clusters in a ~7.7 Mb genome bin). A range of cluster densities was apparent in the rest of the bins. Notably, ladderane biosynthesis, a hallmark of the Planctomycetes, was detected by antiSMASH in all eight of the Planctomycete assemblies (S7 Table), confirming that these are all true Planctomycete genomes. AntiSMASH results show a rich diversity of secondary metabolites in the anammox genomes. Specifically enriched are fatty acids, saccharides, bacteriocins, and terpenes. The OM190 genome was additionally enriched in non-ribosomal peptide synthases, and anatoxin production was predicted. While anatoxin is known to come from cyanobacteria and not from Planctomycetes, its known biosynthetic pathway invovles polyketide synthases, of which 18 are predicted by antiSMASH in this genome. Thus, while this cluster does not likely encode a cyanotoxin, the biosynthetic potential of this genome could certainly produce toxic secondary metabolites. Indeed, a large number of the predicted secondary metabolites are biologically active molecules that may target other cells in the microbial community and could potentially have side effects on mammals.

We saw evidence of rich secondary metabolite biosynthetic potential in several other genomes as well. including representatives of OP3, OP11, Acidobacteria, Bacteroidales, Chlorobi, *Chloroflexi*, *Domibacillus*, *Entotheonella*, *Leptonema*, *Nitrospira*, *Sphingomonas*, *Spirochaetes*, and from DOM were enriched. Notably, we assembled an incomplete genome that appears to be related to cyanobacterial toxin producers. Its best RAPSEARCH hit was to a *Planktothrix aghardii* genome. The 500 kb fragment (MW6-07) is rich in non-ribosomal peptide synthases, which are another toxin production system in the cyanobacteria and can poison humans. In order to confirm whether this might be a toxin producer, we built a BLAST database of microcystin genes found on NCBI and compared to the genome fragment using TBLASTX. We found numerous hits > 300 bp throughout the fragment, but the percent identity was roughly 40%, indicating that the sequences are diverged.

Overall, antiSMASH predicts an enrichment in biosynthetic clusters with antimicrobial activity including bacteriocins, non-ribosomal peptide synthases, polyketide synthases, and lassopeptides. While many antibiotic compounds may have broad targets or even non-antagonistic effects [48], bacteriocins usually have very specific antibiotic activity, often against closely related strains. The prevalence of predicted bacteriocins in the genomes suggests direct competition between genomes. For example, the Brocadiaceae Planctomycete genomes which co-occur in MW6 are predicted to have on average one bacteriocin per genome, which could be used to compete with the related strains.

## Discussion

### Consequences of nitrogen contamination in aquifers based on metagenomics

Overall we find that the metagenomic communities present in groundwater reflect the measured chemical conditions: we measured high nitrogen and DOC as well as a microbial community largely dominated by nitrifier, denitrifier, and anammox bacteria (Tables 1 and 3). Our analysis revealed strain-level variation within key members of this community as well as the potential for rich biosynthetic capacity. We also found evidence for niche specialization based on analysis of the genetic pathways present (Tables 4 and 5). Such niche specialization between species in an anammox community was recently reported for a partial nitritation anammox reactor in a wastewater treatment plant [27]. We find evidence that a similar microbial community is present in shallow, nitrate rich groundwater, and there are multiple anammox strains within a single well. The prevalence of the anammox genomes at over 10% abundance suggests that these bacteria are major drivers of the natural geochemistry of this environment. An implicit consequence is conversion of ammonium and nitrate into nitrite and N_2_ gas. Additionally, nitrite-dependent anaerobic oxidation of methane (n-damo) may be coupled to anammox in this community, reducing potential greenhouse gas emissions [49].

An important aspect of the present study is that the source of the nitrate is cow manure, which also carries a considerable carbon load that supports microbial metabolism. Nitrates derived from synthetic fertilizers do not carry a carbon source and thus may be associated with a considerably different microbial community. Thus, different sources of nitrate could have different potential for bioremediation.

Furthermore, we must consider the source of the microbial community in the environment. The Central Valley of California was once an extensive wetland, and wetland-associated microbial communities perform nitrifier, denitrifer, n-damo, and anammox reactions. If the source of the community were different, we might expect to see a different set metabolic processes with different implications for water quality and greenhouse gas emissions [50].

### Implications for global nutrient cycling

An overlap in anaerobic nitrogen and sulfur redox reactions was shown by Canfield *et al* [47] in the oxygen minimum zone of the ocean. Our metagenomic data and chemical data indicate the potential for a similar overlap in nitrogen and sulfur cycles in groundwater, with OP11 Microgenomates specifically involved through assimilatory sulfur reduction (Table 5). As shown previously (Table 1), nitrate levels were highest in MW5 (106 ppm), and lower in MW6 (21.5 ppm) and DOM (4.3 ppm). The sulfate levels follow a similar trend: MW5, 68.8 ppm; MW6, 15.3 ppm; DOM 2.3 ppm. The microbial abundances (Table 3) and corresponding chemical pathway analysis (Table 5) suggest that these pathways overlap in organisms that exist in the appropriate nutrient conditions. Furthermore the presence of Candidatus *Methylomirabilis* with the anammox communities in MW6 and DOM supports the findings of Shen *et al* [15] that denitrification may be coupled to methane oxidation, reducing potential methane emissions of degrading manure.

### Natural remediation of ammonium to N_2_

The high abundance of anammox and associated nitrifier and denitrifier bacteria in the nitrate-rich samples suggests that excess nitrate and ammonium in groundwater may be naturally remediated [or mineralized] to N_2_ by the endogenous microbiota. The presence of a natural microbial community that closely resembles the nitritation-anammox active sludge community for sewage wastewater denitrification could also be taken as an indication that the shallow groundwater in the Central Valley is recharged from sources similar to sewage wastewater. Based on extensive, controlled studies of this community, e.g [27, 51], it appears possible that simply by decreasing the input of manure into the groundwater, the nitrogen pollutants could decrease below harmful levels. This implication holds true in the shallow groundwater as well as in the deep groundwater, where we still see evidence of the nitritation- anammox community despite lower levels of nitrate (4 ppm). The nitrate:DOC ratio is similar between MW5, MW6, and DOM (≈5), although the total DOC and nitrate levels are an order of magnitude different between each of the samples with MW5>>MW6>>DOM, presumably due to different levels of dilution of the manured water with recharge from the adjacent, unmanured fields. The abundance of a similar nitrifer/denitrifier and anammox microbial community in all three samples appears to mirror the total DOC and nitrate, supporting the notion that bioremediation of nitrate and DOC scales with nutrient abundance both through direct nutrition and through community metabolism [27]. With increased sampling, observed differences in microbial communities may aid in forensic “fingerprinting” approaches [7] to detect sources of nitrate in groundwater [11].

### Groundwater microbiome as a source of bioactive compounds

The metagenomes also indicate a potential concern, which is that the same organisms that remediate the nitrogen also produce bioactive secondary metabolites (e.g. terpenes, toxins, etc) that pose potential health risks and are more difficult and expensive to remove from drinking water. Thus, as groundwater becomes a scarcer and more valuable resource, quantifying the downstream risks of organic manure fertilizer contamination in groundwater becomes a more important priority. There has been speculation about how slow growing anammox bacteria (dividing once per two weeks) can maintain a competitive advantage over faster growing bacteria. The high abundance of secondary metabolite gene clusters in their genomes may give us a clue. Our analysis annotated a diverse array of these gene clusters as various antimicrobials, which could of course help the slow growing anammox cells maintain their dominance in the community. Groundwater microbiomes are unique communities and their metagenomes have not been extensively mined for new biosynthesis pathways. Using antiSMASH we computationally identified many biosynthetic gene clusters that could produce pharmacologically interesting compounds, such as butyrolactone and antibiotics. We suggest the combination of this pharmacological diversity and the unique cell biology of anammox bacteria could make them a fruitful resource for drug discovery.

### Partial assembly rather than short read analysis identifies useful reference genomes

While short read metagenome data can potentially provide insights into taxonomic identities of organisms, we found greatly improved taxonomic inference and functional pathway inference by using partial assembly of the short reads. For instance, while MetaPhlAn analysis gave us a good depiction of the taxonomic similarity between samples (S4 and S5 Figs), the accuracy of assignments was not sufficient to guide the choice of reference genomes for assembly of the whole metagenome deep sequencing reads, indicating that our particular samples have a taxonomic distribution that is poorly represented in the available databases that MetaPhlAn uses.

Assembly of 16S rDNA from short reads is known to be chimera-prone due to the high homology across the tree of life. Solely using EMIRGE to assemble 16S genes and then aligning to SILVA gave us a much more accurate depiction of the phylogenetic diversity in our samples. However, connecting the 16S taxonomy to the genomic bins was problematic. When we tried to link these genes to contigs in the bins using targeted assembly (PRICE), we found that multiple 16S genes assembled to a given genomic bin. While we could make good guesses at which 16S gene belonged to which genomic bin, we could not make these links in an unbiased manner. Therefore, we have omitted them here.

While our analysis reveals only a fraction of the inherent long-tailed distribution of taxa that occur in the groundwater, because we are interested in the major factors shaping water chemistry, the most abundant taxa are the most important to sample. Thus a sequencing depth of ~50 million PE 101 bp reads per sample is quite adequate for assessing the functional geochemistry of groundwater. However, as discussed earlier, a high amount of strain-level variation is present that our current methodologies can only address at a superficial level.

### Strain-level variation in the anammox community

We found evidence for strain-level variation in the anammox community both across samples (e.g. MW5 and MW6) and within bins (e.g. MW5-59_1 and MW5-59_2). While making further distinctions between strains is beyond the scope of this paper, future investigations into the ecological factors that support anammox strain variation with apparently overlapping niches would help define the biology of this globally important denitrifying community. Here we find evidence that at least three related *Brocadiaceae* strains can coexist (e.g. MW6-02, MW6-03 and MW6-13).

### High diversity and abundance of nano-prokaryote genomes in anammox communities

We find many (up to 71 in MW5), highly diverse, nano-prokaryote genomes (S8–S10 Tables), and the abundance of these genomes (as measured by 16S) amounts to over 50% of the community in MW5 (Table 3). Because these organisms have been shown to lack major parts of central metabolism, this observation emphasizes the question posed by Brown et al [2], which is, to what extent do nano-prokaryotes exist as separate cellular entities versus spatially localized to and metabolically dependent upon other cells [52]? Of note is the presence in the small genomes of many partial pathways that affect cellular decision-making (Table 6, S13 Table). In particular, most of the small genomes encode homologs of flagellar chemotaxis components, which we speculate could serve to modify the cellular decision-making behavior of larger cells.

We note that the greater diversity of Chloroflexi, CPR, and DPANN taxa in MW5 versus MW6 and DOM corresponds to a greater presence of nitrate, sulfate, and DOC, which is contrary to macroecological theory and empirical results that demonstrate loss of diversity with increased nutrients [53]. Future studies could address whether these phylogenetic abundance patterns are directly tied to particular nutrients or an indirect consequence of trophic community metabolism, which could aid in optimizing ecology of wastewater treatment bioreactors.

## Conclusions

Our results provide baseline data on the metagenome of nitrate-rich groundwater and reveal abundant and diverse anammox bacteria as well as archaeal and bacterial nano-cells. This study expands the Planctomycete genomic diversity and provides a resource for further investigations of anammox biology. We found a surprising richness of antimicrobial secondary metabolite-encoding gene clusters in the genomes of these bacteria, which could help explain their ability to compete with faster growing species. The study also has important biogeochemical implications for the use of manure as fertilizer due to the scale of agricultural operations using these practices.

## Methods

### Sample sites

Sampling was performed in July 2012. All samples sites were selected in agreement with the property owner. Exact geographic location and identity of the property owner are kept confidential to protect the owner and the dairy. We made individual samples of four water sources on July 11, 2012 at a single 1500 cow dairy in Stanislaus County, California. The three wells and the surface lagoon are all located within 500 m of each other horizontally [10]. Groundwater water levels are 4.3 m below surface. Flow in the area has been estimated at 0.3 m/day laterally based on a hydraulic gradient of 0.001 m/m and estimated hydraulic conductivity of about 75 m/d and an effective transport porosity of 0.25 [22].

The first sample site is the domestic (DOM) water supply for the dairy, a ≈100 m deep well that supplies the drinking water for the cows and human occupants of the dairy. The domestic well had a sealed casing. We sampled the DOM well by connecting a bleached and autoclaved 20 L collection container directly to a tap on the well using a groundwater sampling method previously described [15].

The second sample site is the effluent lagoon (LAG) where all of the cow waste (e.g. feces and urine) is collected to allow concentration, decomposition, and settling out of particulates. The lagoon was sampled by collecting water that was being pumped onto an adjacent corn field. The wastewater effluent lagoon is roughly 30 m by 60 m and ranges in depth over the course of the year but was roughly 1 m deep when we sampled.

The third and fourth sample sites are monitoring wells (MW5 and MW6) drilled adjacent to a corn field that provides silage for cow feed (Fig 1). Water from the effluent lagoon is pumped onto the corn fields in order to provide nitrogen fertilizer. The monitoring wells were constructed of 2-inch diameter PVC with screens open to the surrounding aquifer from 3m to 10m bgs. The monitoring wells were sampled by inserting a pump to 4.3 m bgs and pumping directly into a sealed 20L collection container that was bleached and autoclaved prior to sampling [15]. The monitoring wells were pumped until temperature, electrical conductivity, and pH stabilized or a minimum of 3 casing volumes before connecting to the collection container [54]. The pumping rate was kept between 2 and 5 gallons per minute so that we did not collect water from higher and lower depths.

### Pumping and dialysis filtration scheme

Particulates in the collected water were concentrated by tangential flow filtration [12, 55] using a peristaltic pump recirculating the water through a dialysis filter cartridge (Optiflux, Fresenius Inc). The collected water was concentrated to 300 mL and the filtrate was stored on ice until DNA extraction 12 hours later.

### DNA extraction

DNA extraction was performed using the MO-Bio PowerSoil PowerLyzer kit with the following specifications. For each sample, 100 mL of filtrate was filtered through a 0.22 μm vacuum filter. The filter membrane was then cut into ~1 cm pieces and placed in the manufacturer-supplied bead beating tube. All sample purification steps were performed and then the sample was bound to the silica column, washed, and eluted from the column into 200 μL of TE. The sample was then RNAse A treated and concentrated to 10 μL using the Zymo DCC-5 kit. DNA was quantified using a nanodrop and the concentration was normalized to 5 ng/μL. The concentration was then verified using a Qubit.

For the DOM sample, 108 L of water was concentrated by tangential flow filtration down to a volume of 300 mL. DNA extraction yielded 50 ng. For the LAG sample, 1 mL of water yielded 2,500 ng of DNA. 34 L of MW5 sample was concentrated by tangential flow filtration into 500 mL. 200 mL of this sample was extracted for DNA, yielding ~1000 ng. 64 L of the MW6 sample was concentrated by tangential flow filtration into 500 mL. 200 mL of this sample was extracted for DNA, yielding ~1000 ng.

### Library prep

Deep sequencing libraries were prepared using the Nextera XT DNA Library Prep Kit (Illumina). Each DNA sample was adjusted to 5 ng/μL concentration. For each library, 4 μL DNA was added to 5 μL TD buffer and 1 μL of enzyme. The reaction was heated to 55°C for 5 minutes and then purified on a DCC-5 column and eluted into 12 μL of water. 11 μL of this eluate was loaded as template into a PCR reaction using the KAPA HiFi Library Amplification Kit (Kapa Biosystems). A 30 μL reaction was prepared with 1 μL of each adapter (i.e. i5 and i7 barcodes) primer (adapter oligo stock at 0.5 pmol/μL) and 1 μL of each Solexa primer (i.e. universal Illumina library primers) with the primer stock at 10 pmol/μL. The reaction was run with the following parameters: 72°C 3 min, 98°C 30 sec, and 12 cycles of (98°C 10 sec, 63°C 30 sec, 72°C 3 min). The libraries were then size selected for a 450 to 600 bp length smear using the LabChipXT (Caliper).

### Whole metagenome shotgun sequencing

The Illumina libraries from each water sample were pooled and sequenced on an Illumina HiSeq 2000 using a 101 bp paired-end read length and dual 6 bp index reads. Clustering on the flow cell and sequencing was performed by the UCSF Mission Bay sequencing core facility.

We sequenced each library on 1/3 of an Illumina HiSeq 2000 flow cell using 101 bp paired-end (PE) sequencing (S11 and S12 Tables). All sequences are available from the NCBI Sequence Read Archive under BioProject PRJNA342017. https://trace.ncbi.nlm.nih.gov/Traces/study/?acc=SRP090828

### Bioinformatics analysis

A complete list of all bioinformatics software, versions, and parameters with launch scripts is included in S16 Table. A description of the usage is provided here. Quality filtering was performed using PrinSeq [56], and reads were merged using USEARCH8 [57].

### Targeted 16S assembly and phylogenetic analysis

We used EMIRGE [23] to assemble the most-abundant 16S genes present in our samples. Phylogenetic analysis was performed using ARB [58] and the SILVA database [59, 60].

### Metagenome assembly and genome binning

We then performed a non-exhaustive, high confidence, metagenomic assembly for each sample using IDBA_UD [28], and we used REAPR [61] to break contigs at any places with non-conformant fragment coverage distribution (FCD). Next, we binned contigs by kmer distribution using VizBin [30] with the kmer size set to 5 nucleotides and a minimum contig length of 1000 bp.

### Genomic bin validation and refinement

Rough taxonomic identity of the genomic bins was assessed using RAPSEARCH [25] and the UniProt UniRef100 database [26]. We assessed the coverage of the individual contigs in the bins using Bowtie2. The coverage data was converted to RPKM values and a histogram of the mean contig coverages was plotted for each bin. Bins with multi-modal coverage were split based on coverage and then reprocessed using RAPSEARCH. CheckM [33] was used to calculate bin completeness, contamination with other species, and existence of multiple strains of the same species. The metrics were assessed both using CheckM’s own databases as well as using a conserved single copy gene database of 111 genes (github.com/MadsAlbertsen/mmgenome/tree/master/scripts). Genomic bin extension was performed for selected anammox and denitrifying genomes using targeted assembly mode in PRICE [32]. Misassemblies were detected using REAPR, and contigs were broken at these misassembly points. The validation and refinement process was repeated iteratively until bin quality was sufficient for our purposes.

### Evaluation of biosynthetic potential

We first performed gene calling and annotation using Prokka [62] with Prodigal [63]. First, the coding sequences were then aligned to the KEGG database of prokaryotic orthologous genes using BLASTP. Results were converted to KEGG Mapper files and visualized using the KEGG Mapper website (http://www.genome.jp/kegg/tool/map_pathway.html). Next, the Genbank files generated by Prokka were loaded into antiSMASH 2.0 [64]. Results for both methods were manually complied into the tables presented. Pathway coverage cutoffs: high >80%; moderate >50%; sparse <50%.

## Supporting information

S1 Fig Planctomycete bin MW6-09 has best (but low) homology to the *Singulisphaera acidiphilus*. BRIG was used to align available Planctomycete reference genomes to the MW6-09 bin by blastn. Radial colored bars in the concentric rings indicate nucleotide homology (30–100%). See graphical legend for ring identities. Contig order is that of the MW6-09 genome. The best coverage is seen for *Singulisphaera*, but the coverage is still low, consistent with MW6-09 representing OM190, as the 16S data suggest.
(TIF)

S2 Fig VizBin clustering of the Planctomycete genomic bins shows evidence of at least 6 distinct genomes. See graphical legend to determine identity of each cluster. MW5-59_1 and MW5-59_2 may be strain variants because they overlap in their pentanucleotide distribution clustering but have distinct relative abundances (S1 Table). MW6-13 also overlaps the MW5-59 bins and may likewise be multiple strains of the same species.
(TIF)

S3 Fig Homology of assembled Brocadiaceae genomes to the *Brocadia sinica* reference genome. BRIG was used to compare the Brocadiaceae genomic bins to the reference *Jettenia caeni* by blastn. Radial colored bars in the concentric rings indicate nucleotide homology (30–100%). See graphical legend for ring identities. MW5 bins are shown in green, MW6 in red, and DOM in blue. Contig order is that of the reference genome.
(TIF)

S4 Fig MetaPhlAn 1.0 analysis of short read data. MetaPhlAn 1.0 analysis of the short reads to estimate taxonomic relative abundance at the genus level. The color scale bar indicates the percentage of reads aligning to the indicated taxon reference sequences.
(PDF)

S5 Fig MetaPhlAn 2.0 analysis of short read data. MetaPhlAn 2.0 analysis of the short reads to estimate taxonomic relative abundance at the genus level. The color scale bar indicates the percentage of reads aligning to the indicated taxon reference sequences.
(PDF)

S1 Table MW5 genomic bin statistics including taxonomic calls, coverage, assembly metrics, and completeness estimates. (XLSX)

S2 Table MW6 genomic bin statistics including taxonomic calls, coverage, assembly metrics, and completeness estimates. (XLSX)

S3 Table DOM genomic bin statistics including taxonomic calls, coverage, assembly metrics, and completeness estimates. (XLSX)

S4 Table LAG genomic bin statistics including taxonomic calls, coverage, assembly metrics, and completeness estimates. (XLSX)

S5 Table Comparison of the taxonomic composition of the communities described here with the community of a wastewater treatment plant described by Speth et al. (XLSX)

S6 Table KEGG pathway and module coverage by water sample. (PDF)

S7 Table AntiSMASH cluster predictions by genomic bin in each water sample. (XLSX)

S8 Table MW5 genomic bin basic statistics for initial unrefined bins. (XLSX)

S9 Table MW6 genomic bin basic statistics for initial unrefined bins. (XLSX)

S10 Table DOM genomic bin basic statistics for initial unrefined bins. (XLSX)

S11 Table Number FASTQ sequence reads passing QC. (DOCX)

S12 Table Total bp for FASTQ sequences for reads passing QC. (DOCX)

S13 Table Complete version of Table 5, including transporters. (XLSX)

S14 Table Water chemistry data with standard error included to supplement Table 1. (XLSX)

S15 Table ICP-MS data with standard error included to supplement Table 2. (XLSX)

S16 Table Bioinformatics software used. (DOCX)
