# Supplementary figures and images for: Assessing biosynthetic potential of agricultural groundwater through metagenomic sequencing: A diverse anammox community dominates nitrate-rich groundwater
Source: PLoS One. 2017 Apr 6;12(4):e0174930. doi: 10.1371/journal.pone.0174930 (PMC5383146; doi:10.1371/journal.pone.0174930)

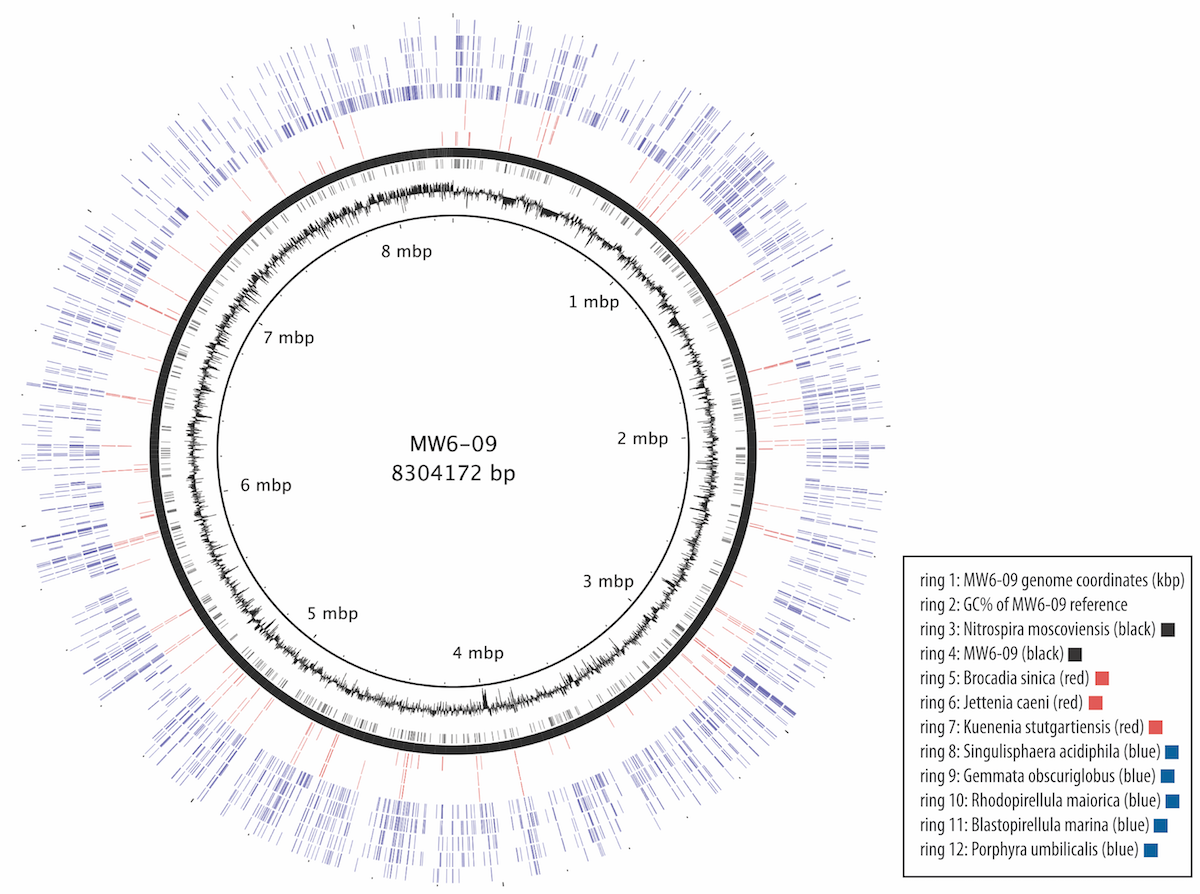

Supplement: S1 Fig — BRIG was used to align available Planctomycete reference genomes to the MW6-09 bin by blastn. Radial colored bars in the concentric rings indicate nucleotide homology (30–100%). See graphical legend for ring identities. Contig order is that of the MW6-09 genome. The best coverage is seen for Singulisphaera, but the coverage is still low, consistent with MW6-09 representing OM190, as the 16S data suggest. (TIF) [file pone.0174930.s001.tif]

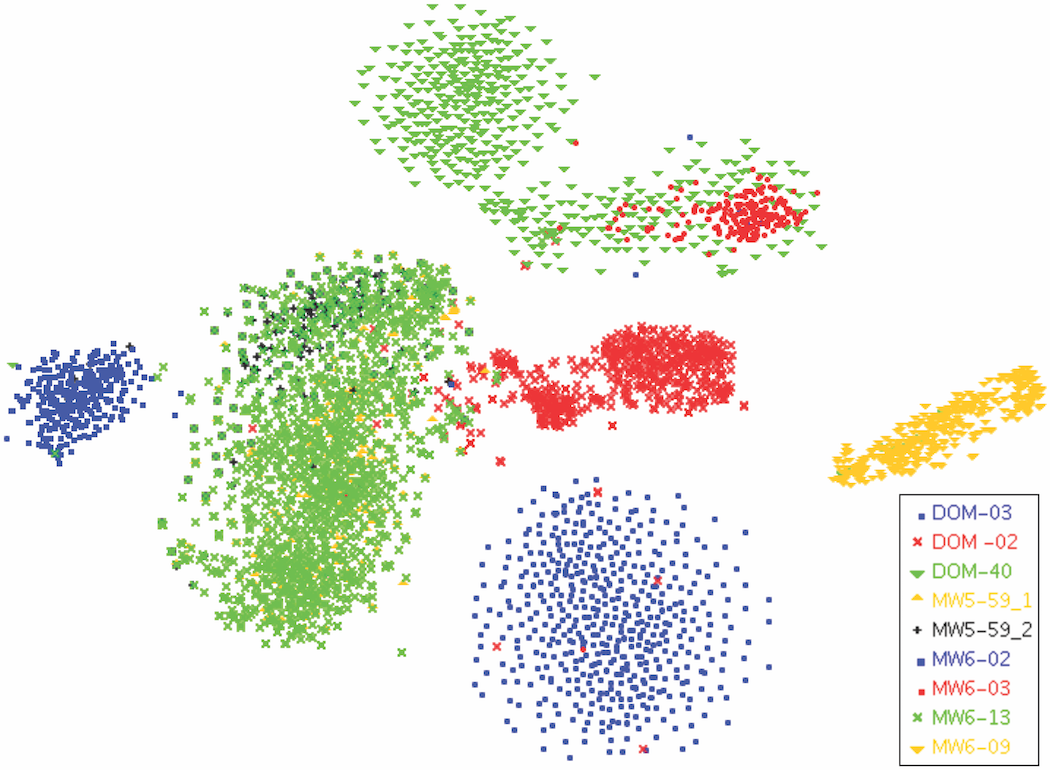

Supplement: S2 Fig — See graphical legend to determine identity of each cluster. MW5-59_1 and MW5-59_2 may be strain variants because they overlap in their pentanucleotide distribution clustering but have distinct relative abundances (S1 Table). MW6-13 also overlaps the MW5-59 bins and may likewise be multiple strains of the same species. (TIF) [file pone.0174930.s002.tif]

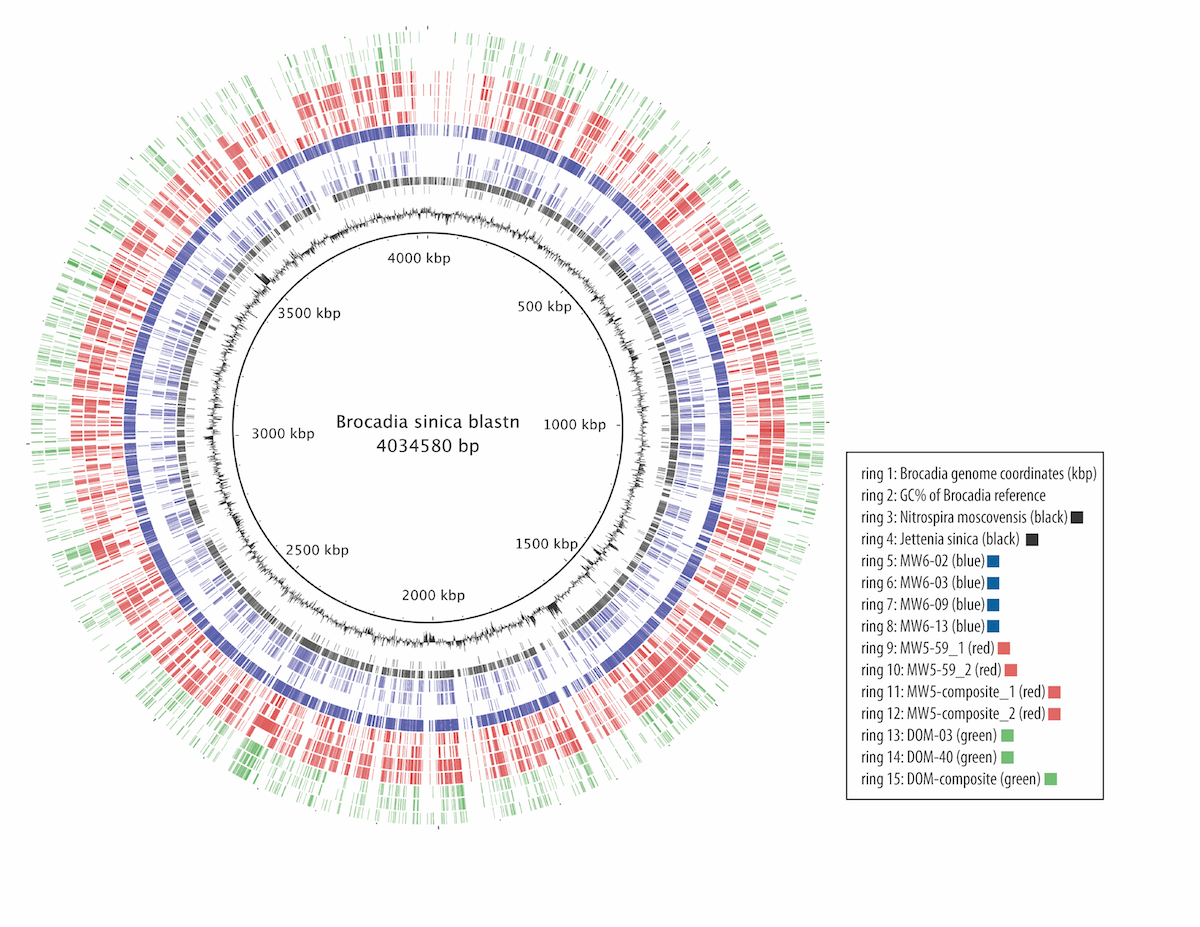

Supplement: S3 Fig — BRIG was used to compare the Brocadiaceae genomic bins to the reference Jettenia caeni by blastn. Radial colored bars in the concentric rings indicate nucleotide homology (30–100%). See graphical legend for ring identities. MW5 bins are shown in green, MW6 in red, and DOM in blue. Contig order is that of the reference genome. (TIF) [file pone.0174930.s003.tif]

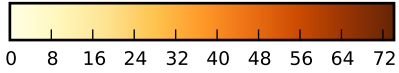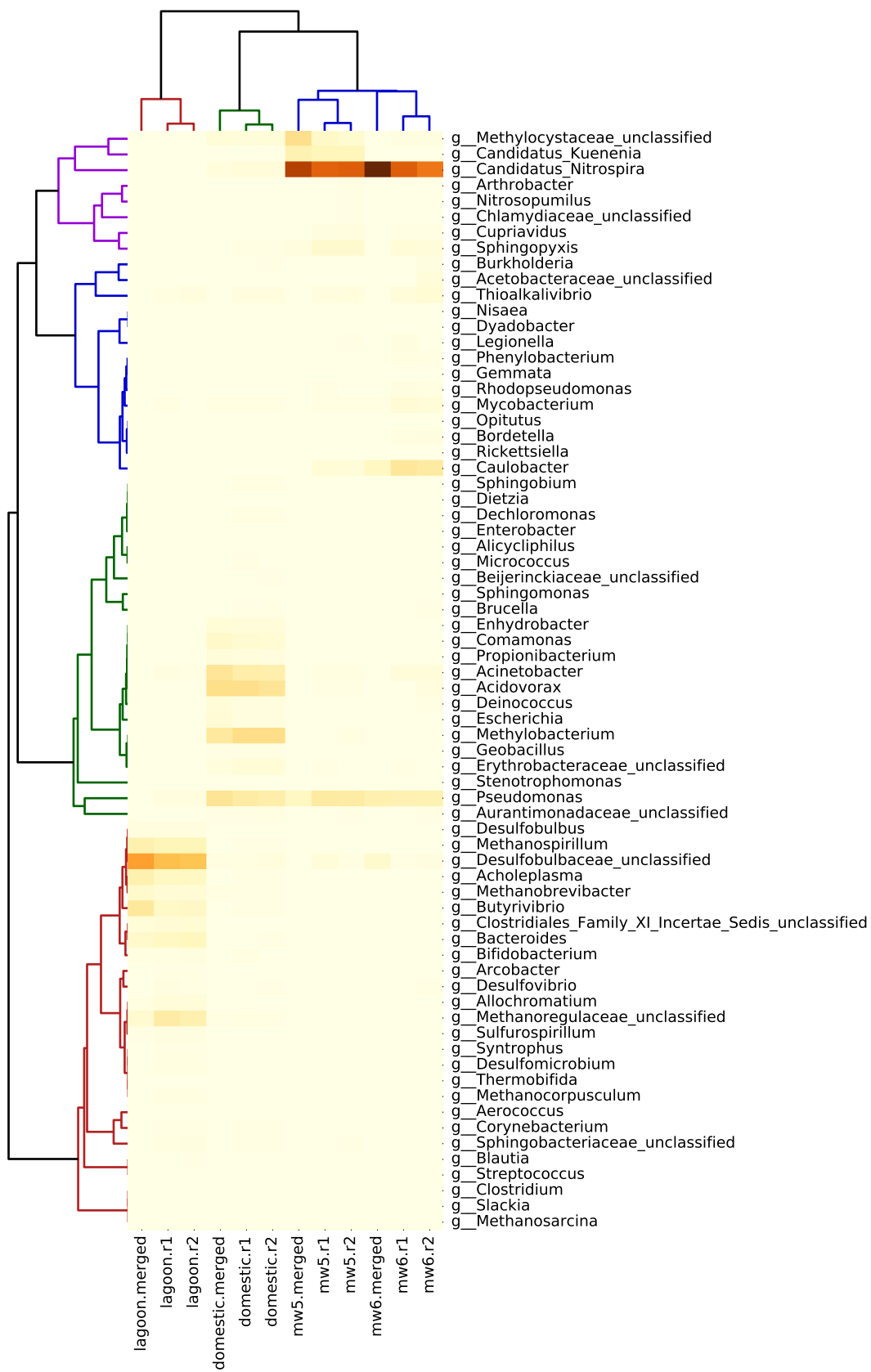

Supplement: S4 Fig — MetaPhlAn 1.0 analysis of the short reads to estimate taxonomic relative abundance at the genus level. The color scale bar indicates the percentage of reads aligning to the indicated taxon reference sequences. (PDF) [file pone.0174930.s004.pdf]

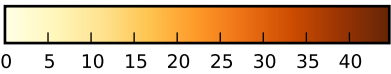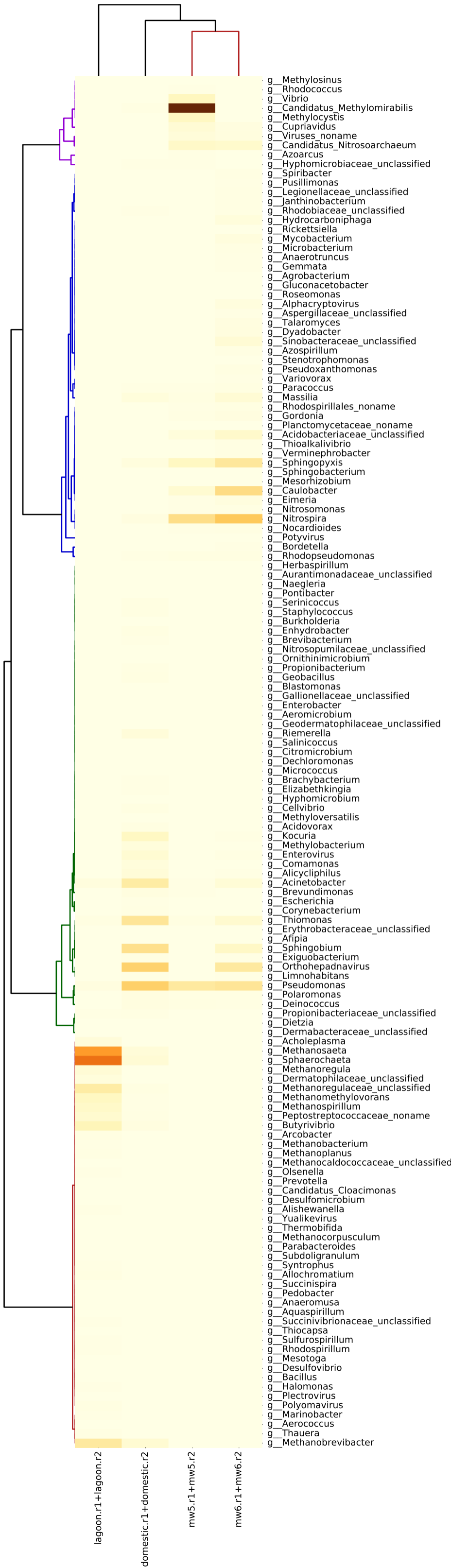

Supplement: S5 Fig — MetaPhlAn 2.0 analysis of the short reads to estimate taxonomic relative abundance at the genus level. The color scale bar indicates the percentage of reads aligning to the indicated taxon reference sequences. (PDF) [file pone.0174930.s005.pdf]
