# Supplementary material for: Assessing biosynthetic potential of agricultural groundwater through metagenomic sequencing: A diverse anammox community dominates nitrate-rich groundwater
Source: PLoS One. 2017 Apr 6;12(4):e0174930. doi: 10.1371/journal.pone.0174930 (PMC5383146; doi:10.1371/journal.pone.0174930)
Supplement: S6 Table — (PDF) [file pone.0174930.s011.pdf]

Table S6: KEGG module coverage by water sample

| pathway | name | module | name | domestic | lagoon | mw5 | mw6 |
|---|---|---|---|---|---|---|---|
| map00010 | Glycolysis / Gluconeogenesis | M00001 | Glycolysis (Embden-Meyerhof pathway), glucose => pyruvate | | | | |
| | | M00003 | Gluconeogenesis, oxaloacetate => fructose-6P | | | | |
| | | M00114 | Ascorbate biosynthesis, plants, glucose-6P => ascorbate | | | | |
| | | M00265 | PTS system, glucose-specific II component | | | | |
| | | M00266 | PTS system, maltose and glucose-specific II component | | | | |
| | | M00268 | PTS system, arbutin-like II component | | | | |
| | | M00272 | PTS system, arbutin-, cellobiose-, and salicin-specific II component | | | | |
| | | M00307 | Pyruvate oxidation, pyruvate => acetyl-CoA | | | | |
| map00020 | Citrate cycle (TCA cycle) | M00003 | Gluconeogenesis, oxaloacetate => fructose-6P | | | | |
| | | M00009 | Citrate cycle (TCA cycle, Krebs cycle) | | | | |
| | | M00011 | Citrate cycle, second carbon oxidation, 2-oxoglutarate => oxaloac... | | | | |
| map00030 | Pentose phosphate pathway | M00004 | Pentose phosphate pathway (Pentose phosphate cycle) | | | | |
| | | M00006 | Pentose phosphate pathway, oxidative phase, glucose 6P => ribul... | | | | |
| | | M00008 | Entner-Doudoroff pathway, glucose-6P => glyceraldehyde-3P + p... | | | | |
| | | M00061 | D-Glucuronate degradation, D-glucuronate => pyruvate + D-glycer... | | | | |
| | | M00308 | Semi-phosphorylative Entner-Doudoroff pathway, gluconate => gl... | | | | |
| | | M00309 | Non-phosphorylative Entner-Doudoroff pathway, gluconate/galact... | | | | |
| | | M00631 | D-Galacturonate degradation (bacteria), D-galacturonate => pyruv... | | | | |
| | | M00633 | Semi-phosphorylative Entner-Doudoroff pathway, gluconate/galac... | | | | |
| map00040 | Pentose and glucuronate interconversions | M00014 | Glucuronate pathway (uronate pathway) | | | | |
| | | M00061 | D-Glucuronate degradation, D-glucuronate => pyruvate + D-glycer... | | | | |
| | | M00081 | Pectin degradation | | | | |
| | | M00129 | Ascorbate biosynthesis, animals, glucose-1P => ascorbate | | | | |
| | | M00630 | D-Galacturonate degradation (fungi), D-galacturonate => glycerol | | | | |
| | | M00631 | D-Galacturonate degradation (bacteria), D-galacturonate => pyruv... | | | | |
| map00051 | Fructose and mannose metabolism | M00114 | Ascorbate biosynthesis, plants, glucose-6P => ascorbate | | | | |
| | | M00273 | PTS system, fructose-specific II component | | | | |
| | | M00274 | PTS system, mannitol-specific II component | | | | |
| | | M00276 | PTS system, mannose-specific II component | | | | |
| | | M00278 | PTS system, sorbose-specific II component | | | | |
| | | M00280 | PTS system, glucitol/sorbitol-specific II component | | | | |
| map00052 | Galactose metabolism | M00277 | PTS system, N-acetylgalactosamine-specific II component | | | | |
| | | M00279 | PTS system, galactitol-specific II component | | | | |
| | | M00281 | PTS system, lactose-specific II component | | | | |
| | | M00287 | PTS system, galactosamine-specific II component | | | | |
| | | M00552 | D-galactonate degradation, De Ley-Doudoroff pathway, D-galacto... | | | | |
| | | M00554 | Nucleotide sugar biosynthesis, galactose => UDP-galactose | | | | |
| | | M00632 | Galactose degradation, Leloir pathway, galactose => alpha-D-gluc... | | | | |
| map00053 | Ascorbate and aldarate metabolism | M00129 | Ascorbate biosynthesis, animals, glucose-1P => ascorbate | | | | |
| | | M00283 | PTS system, ascorbate-specific II component | | | | |
| | | M00550 | Ascorbate degradation, ascorbate => D-xylulose-5P | | | | |
| map00061 | Fatty acid biosynthesis | M00082 | Fatty acid biosynthesis, initiation | | | | |
| | | M00083 | Fatty acid biosynthesis, elongation | | | | |
| map00062 | Fatty acid elongation | M00085 | Fatty acid biosynthesis, elongation, mitochondria | | | | |
| | | M00415 | Fatty acid biosynthesis, elongation, endoplasmic reticulum | | | | |
| map00071 | Fatty acid metabolism | M00086 | beta-Oxidation, acyl-CoA synthesis | | | | |
| | | M00087 | beta-Oxidation | | | | |
| map00072 | Synthesis and degradation of ketone bodies | M00088 | Ketone body biosynthesis, acetyl-CoA => acetoacetate/3-hydroxy... | | | | |
| map00100 | Steroid biosynthesis | M00101 | Cholesterol biosynthesis, squalene 2,3-epoxide => cholesterol | | | | |
| | | M00102 | Ergocalciferol biosynthesis | | | | |
| | | M00103 | Cholecalciferol biosynthesis | | | | |
| map00120 | Primary bile acid biosynthesis | M00104 | Bile acid biosynthesis, cholesterol => cholate/chenodeoxycholate | | | | |
| | | M00106 | Conjugated bile acid biosynthesis, cholate => taurocholate/glycoc... | | | | |
| map00130 | Ubiquinone and other terpenoid-quinone bi... | M00112 | Tocopherol/tocotorienol biosynthesis | | | | |
| | | M00116 | Menaquinone biosynthesis, chorismate => menaquinone | | | | |
| | | M00117 | Ubiquinone biosynthesis, prokaryotes, chorismate => ubiquinone | | | | |
| | | M00128 | Ubiquinone biosynthesis, eukaryotes, 4-hydroxybenzoate => ubiq... | | | | |
| map00140 | Steroid hormone biosynthesis | M00107 | Steroid hormone biosynthesis, cholesterol => prognenolone => pr... | | | | |
| | | M00108 | C21-Steroid hormone biosynthesis, progesterone => corticosteron... | | | | |
| | | M00109 | C21-Steroid hormone biosynthesis, progesterone => cortisol/corti... | | | | |
| | | M00110 | C19/C18-Steroid hormone biosynthesis, pregnenolone => androst... | | | | |
| map00190 | Oxidative phosphorylation | M00142 | NADH:ubiquinone oxidoreductase, mitochondria | | | | |
| | | M00143 | NADH dehydrogenase (ubiquinone) Fe-S protein/flavoprotein com... | | | | |
| | | M00144 | NADH:quinone oxidoreductase, prokaryotes | | | | |
| | | M00145 | NAD(P)H:quinone oxidoreductase, chloroplasts and cyanobacteria | | | | |
| | | M00146 | NADH dehydrogenase (ubiquinone) 1 alpha subcomplex | | | | |
| | | M00147 | NADH dehydrogenase (ubiquinone) 1 beta subcomplex | | | | |
| | | M00148 | Succinate dehydrogenase (ubiquinone) | | | | |
| | | M00149 | Succinate dehydrogenase, prokaryotes | | | | |
| | | M00150 | Fumarate reductase, prokaryotes | | | | |
| | | M00151 | Cytochrome bc1 complex respiratory unit | | | | |
| | | M00152 | Cytochrome bc1 complex | | | | |
| | | M00153 | Cytochrome d ubiquinol oxidase | | | | |
| | | M00154 | Cytochrome c oxidase | | | | |
| | | M00155 | Cytochrome c oxidase, prokaryotes | | | | |
| | | M00156 | Cytochrome c oxidase, cbb3-type | | | | |
| | | M00157 | F-type ATPase, prokaryotes and chloroplasts | | | | |
| | | M00158 | F-type ATPase, eukaryotes | | | | |
| | | M00159 | V/A-type ATPase, prokaryotes | | | | |
| | | M00160 | V-type ATPase, eukaryotes | | | | |
| | | M00416 | Cytochrome aa3-600 menaquinol oxidase | | | | |
| | | M00417 | Cytochrome o ubiquinol oxidase | | | | |
| map00195 | Photosynthesis | M00157 | F-type ATPase, prokaryotes and chloroplasts | | | | |
| | | M00161 | Photosystem II | | | | |
| | | M00162 | Cytochrome b6f complex | | | | |
| | | M00163 | Photosystem I | | | | |
| map00230 | Purine metabolism | M00048 | Inosine monophosphate biosynthesis, PRPP + glutamine => IMP | | | | |
| | | M00049 | Adenine ribonucleotide biosynthesis, IMP => ADP,ATP | | | | |
| | | M00050 | Guanine ribonucleotide biosynthesis IMP => GDP,GTP | | | | |
| | | M00546 | Purine degradation, xanthine => urea | | | | |
| map00240 | Pyrimidine metabolism | M00046 | Pyrimidine degradation, uracil => beta-alanine, thymine => 3-amin... | | | | |
| | | M00051 | Uridine monophosphate biosynthesis, glutamine (+ PRPP) => UMP | | | | |
| | | M00052 | Pyrimidine ribonucleotide biosynthesis, UMP => UDP/UTP,CDP... | | | | |
| | | M00053 | Pyrimidine deoxyribonuleotide biosynthesis, CDP/CTP => dCDP/d... | | | | |
| map00250 | Alanine, aspartate and glutamate metabo... | M00027 | GABA (gamma-Aminobutyrate) shunt | | | | |
| map00260 | Glycine, serine and threonine metabolism | M00018 | Threonine biosynthesis, aspartate => homoserine => threonine | | | | |
| | | M00555 | Betaine biosynthesis, choline => betaine | | | | |
| map00270 | Cysteine and methionine metabolism | M00017 | Methionine biosynthesis, apartate => homoserine => methionine | | | | |
| | | M00034 | Methionine salvage pathway | | | | |
| | | M00035 | Methionine degradation | | | | |
| | | M00338 | Cysteine biosynthesis, homocysteine + serine => cysteine | | | | |
| | | M00368 | Ethylene biosynthesis, methionine => ethylene | | | | |
| | | M00609 | Cysteine biosynthesis, methionine => cysteine | | | | |
| map00280 | Valine, leucine and isoleucine degradation | M00036 | Leucine degradation, leucine => acetoacetate + acetyl-CoA | | | | |
| map00290 | Valine, leucine and isoleucine biosynthesis | M00570 | Isoleucine biosynthesis, threonine => 2-oxobutanoate => isoleucine | | | | |
| map00300 | Lysine biosynthesis | M00016 | Lysine biosynthesis, succinyl-DAP pathway, aspartate => lysine | | | | |
| | | M00030 | Lysine biosynthesis, AAA pathway, 2-oxoglutarate => 2-aminoadi... | | | | |
| | | M00525 | Lysine biosynthesis, acetyl-DAP pathway, aspartate => lysine | | | | |
| | | M00526 | Lysine biosynthesis, DAP dehydrogenase pathway, aspartate => l... | | | | |
| | | M00527 | Lysine biosynthesis, DAP aminotransferase pathway, aspartate =... | | | | |
| map00310 | Lysine degradation | M00032 | Lysine degradation, lysine => saccharopine => acetoacetyl-CoA | | | | |
| map00311 | Penicillin and cephalosporin biosynthesis | M00672 | Penicillin biosynthesis, aminoadipate + cycteine + valine => penici... | | | | |
| | | M00673 | Cephamycin C biosynthesis, aminoadipate + cycteine + valine => ... | | | | |
| map00330 | Arginine and proline metabolism | M00015 | Proline biosynthesis, glutamate => proline | | | | |
| | | M00029 | Urea cycle | | | | |
| | | M00047 | Creatine pathway | | | | |
| | | M00133 | Polyamine biosynthesis, arginine => agmatine => putrescine => s... | | | | |
| | | M00134 | Polyamine biosynthesis, arginine => ornithine => putrescine | | | | |
| | | M00135 | GABA biosynthesis, eukaryotes, putrescine => GABA | | | | |
| | | M00136 | GABA biosynthesis, prokaryotes, putrescine => GABA | | | | |
| map00331 | Clavulanic acid biosynthesis | M00674 | Clavaminate biosynthesis, arginine + glyceraldehyde-3P => clava... | | | | |
| map00332 | Carbapenem biosynthesis | M00675 | Carbapenem-3-carboxylate biosynthesis, pyrroline-5-carboxylate ... | | | | |
| map00340 | Histidine metabolism | M00026 | Histidine biosynthesis, PRPP => histidine | | | | |
| | | M00045 | Histidine degradation, histidine => N-formiminoglutamate => gluta... | | | | |
| map00350 | Tyrosine metabolism | M00042 | Catecholamine biosynthesis, tyrosine => dopamine => noradrenal... | | | | |
| | | M00043 | Thyroid hormone biosynthesis, tyrosine => triiodothyronine/thyro... | | | | |
| | | M00044 | Tyrosine degradation, tyrosine => homogentisate | | | | |
| | | M00533 | Homoprotocatechuate degradation, homoprotocatechuate => 2-ox... | | | | |
| map00360 | Phenylalanine metabolism | M00350 | Capsaicin biosynthesis, L-Phenylalanine => Capsaicin | | | | |
| | | M00545 | Trans-cinnamate degradation, trans-cinnamate => acetyl-CoA | | | | |
| map00362 | Benzoate degradation | M00540 | Benzoate degradation, cyclohexanecarboxylic acid =>pimeloyl-CoA | | | | |
| | | M00541 | Benzoyl-CoA degradation, benzoyl-CoA => 3-hydroxypimeloyl-CoA | | | | |
| | | M00547 | Benzene/toluene degradation, benzene => catechol / toluene => 3... | | | | |
| | | M00548 | Benzene degradation, benzene => catechol | | | | |
| | | M00551 | Benzoate degradation, benzoate => catechol / methylbenzoate =>... | | | | |
| | | M00568 | Catechol ortho-cleavage, catechol => 3-oxoadipate | | | | |
| | | M00569 | Catechol meta-cleavage, catechol => acetyl-CoA / 4-methylcatech... | | | | |
| map00380 | Tryptophan metabolism | M00037 | Melatonin biosynthesis, tryptophan => serotonin => melatonin | | | | |
| | | M00038 | Tryptophan metabolism, tryptophan => kynurenine => 2-aminomu... | | | | |
| map00400 | Phenylalanine, tyrosine and tryptophan bio... | M00022 | Shikimate pathway, phosphoenolpyruvate + erythrose-4P => chori... | | | | |
| | | M00023 | Tryptophan biosynthesis, chorismate => tryptophan | | | | |
| | | M00024 | Phenylalanine biosynthesis, chorismate => phenylalanine | | | | |
| | | M00025 | Tyrosine biosynthesis, chorismate => tyrosine | | | | |
| | | M00040 | Tyrosine biosynthesis, prephanate => pretyrosine => tyrosine | | | | |
| map00403 | Indole diterpene alkaloid biosynthesis | M00661 | Paspaline biosynthesis, geranylgeranyl-PP + indoleglycerol phosp... | | | | |
| map00460 | Cyanoamino acid metabolism | M00369 | Cyanogenic glycoside biosynthesis, tyrosine => dhurrin | | | | |
| map00480 | Glutathione metabolism | M00118 | Glutathione biosynthesis, glutamate => glutathione | | | | |
| map00500 | Starch and sucrose metabolism | M00266 | PTS system, maltose and glucose-specific II component | | | | |
| | | M00269 | PTS system, sucrose-specific II component | | | | |
| | | M00270 | PTS system, trehalose-specific II component | | | | |
| | | M00565 | Trehalose biosynthesis, D-glucose 1P => trehalose | | | | |
| map00510 | N-Glycan biosynthesis | M00055 | N-glycan precursor biosynthesis | | | | |
| | | M00072 | N-glycosylation by oligosaccharyltransferase | | | | |
| | | M00073 | N-glycan precursor trimming | | | | |
| | | M00074 | N-glycan biosynthesis, high-mannose type | | | | |
| | | M00075 | N-glycan biosynthesis, complex type | | | | |
| map00512 | O-Glycan biosynthesis | M00056 | O-glycan biosynthesis, mucin type core | | | | |
| map00513 | High-mannose type N-glycan biosynthesis | M00074 | N-glycan biosynthesis, high-mannose type | | | | |
| map00520 | Amino sugar and nucleotide sugar metabo... | M00267 | PTS system, N-acetylglucosamine-specific II component | | | | |
| | | M00282 | PTS system, D-glucosamine-specific II component | | | | |
| | | M00361 | Nucleotide sugar biosynthesis, eukaryotes | | | | |
| | | M00362 | Nucleotide sugar biosynthesis, prokaryotes | | | | |
| | | M00549 | Nucleotide sugar biosynthesis, glucose => UDP-glucose | | | | |
| | | M00554 | Nucleotide sugar biosynthesis, galactose => UDP-galactose | | | | |
| map00531 | Glycosaminoglycan degradation | M00076 | Dermatan sulfate degradation | | | | |
| | | M00077 | Chondroitin sulfate degradation | | | | |
| | | M00078 | Heparan sulfate degradation | | | | |
| | | M00079 | Keratan sulfate degradation | | | | |
| map00532 | Glycosaminoglycan biosynthesis - chondroi... | M00057 | Glycosaminoglycan biosynthesis, linkage tetrasaccharide | | | | |
| | | M00058 | Glycosaminoglycan biosynthesis, chondroitin sulfate backbone | | | | |
| map00534 | Glycosaminoglycan biosynthesis - heparan ... | M00059 | Glycosaminoglycan biosynthesis, heparan sulfate backbone | | | | |
| map00540 | Lipopolysaccharide biosynthesis | M00060 | Lipopolysaccharide biosynthesis, KDO2-lipid A | | | | |
| | | M00063 | CMP-KDO biosynthesis | | | | |
| | | M00064 | ADP-L-glycero-D-manno-heptose biosynthesis | | | | |
| | | M00080 | Lipopolysaccharide biosynthesis, inner core => outer core => O-a... | | | | |
| map00561 | Glycerolipid metabolism | M00089 | Triacylglycerol biosynthesis | | | | |
| | | M00098 | Acylglycerol degradation | | | | |
| map00562 | Inositol phosphate metabolism | M00130 | Inositol phosphate metabolism, PI=> PIP2 => Ins(1,4,5)P3 => Ins(... | | | | |
| | | M00131 | Inositol phosphate metabolism, Ins(1,3,4,5)P4 => Ins(1,3,4)P3 => ... | | | | |
| | | M00132 | Inositol phosphate metabolism, Ins(1,3,4)P3 => phytate | | | | |
| map00563 | Glycosylphosphatidylinositol(GPI)-anchor b... | M00065 | GPI-anchor biosynthesis, core oligosaccharide | | | | |
| map00564 | Glycerophospholipid metabolism | M00090 | Phosphatidylcholine (PC) biosynthesis, choline => PC | | | | |
| | | M00091 | Phosphatidylcholine (PC) biosynthesis, PE => PC | | | | |
| | | M00092 | Phosphatidylethanolamine (PE) biosynthesis, ethanolamine => PE | | | | |
| | | M00093 | Phosphatidylethanolamine (PE) biosynthesis, PA => PS => PE | | | | |
| map00565 | Ether lipid metabolism | M00067 | Sulfoglycolipids biosynthesis, ceramide/1-alkyl-2-acylglycerol => s... | | | | |
| map00592 | alpha-Linolenic acid metabolism | M00113 | Jasmonic acid biosynthesis | | | | |
| map00600 | Sphingolipid metabolism | M00066 | Lactosylceramide biosynthesis | | | | |
| | | M00067 | Sulfoglycolipids biosynthesis, ceramide/1-alkyl-2-acylglycerol => s... | | | | |
| | | M00094 | Ceramide biosynthesis | | | | |
| | | M00099 | Sphingosine biosynthesis | | | | |
| | | M00100 | Sphingosine degradation | | | | |
| map00601 | Glycosphingolipid biosynthesis - lacto and ... | M00070 | Glycosphingolipid biosynthesis, lacto-series, LacCer => Lc4Cer | | | | |
| | | M00071 | Glycosphingolipid biosynthesis, neolacto-series, LacCer => nLc... | | | | |
| map00603 | Glycosphingolipid biosynthesis - globo series | M00068 | Glycosphingolipid biosynthesis, globo-series, LacCer => Gb4Cer | | | | |
| map00604 | Glycosphingolipid biosynthesis - ganglio se... | M00069 | Glycosphingolipid biosynthesis, ganglio series, LacCer => GT3 | | | | |
| map00621 | Dioxin degradation | M00543 | Biphenyl degradation, biphenyl => 2-oxopent-4-enoate + benzoate | | | | |
| | | M00544 | Carbazole degradation, carbazole => 2-oxopent-4-enoate + anthr... | | | | |
| map00622 | Xylene degradation | M00419 | Cymene degradation, p-cymene => p-cumate | | | | |
| | | M00537 | Xylene degradation, xylene => methylbenzoate | | | | |
| | | M00539 | Cumate degradation, p-cumate => 2-oxopent-4-enoate + 2-methyl... | | | | |
| map00623 | Toluene degradation | M00418 | Toluene degradation, anaerobic, toluene => benzoyl-CoA | | | | |
| | | M00538 | Toluene degradation, toluene => benzoate | | | | |
| map00624 | Polycyclic aromatic hydrocarbon degradation | M00623 | Phthalate degradation, phthalate => protocatechuate | | | | |
| | | M00624 | Terephthalate degradation, terephthalate => 3,4-dihydroxybenzoate | | | | |
| | | M00636 | Phthalate degradation, phthalate => protocatechuate | | | | |
| map00626 | Naphthalene degradation | M00534 | Naphthalene degradation, naphthalene => salicylate | | | | |
| | | M00638 | Salicylate degradation, salicylate => gentisate | | | | |
| map00627 | Aminobenzoate degradation | M00637 | Anthranilate degradation, anthranilate => catechol | | | | |
| map00630 | Glyoxylate and dicarboxylate metabolism | M00012 | Glyoxylate cycle | | | | |
| | | M00373 | Ethylmalonyl pathway | | | | |
| | | M00532 | Photorespiration | | | | |
| map00640 | Propanoate metabolism | M00013 | Malonate semialdehyde pathway, propanoyl-CoA => Acetyl-CoA | | | | |
| map00670 | One carbon pool by folate | M00126 | Tetrahydrofolate biosynthesis, GTP => THF | | | | |
| | | M00140 | C1-unit interconversion, prokaryotes | | | | |
| | | M00141 | C1-unit interconversion, eukaryotes | | | | |
| map00680 | Methane metabolism | M00344 | Formaldehyde assimilation, xylulose monophosphate pathway | | | | |
| | | M00345 | Formaldehyde assimilation, ribulose monophosphate pathway | | | | |
| | | M00346 | Formaldehyde assimilation, serine pathway | | | | |
| | | M00356 | Methanogenesis, methanol => methane | | | | |
| | | M00357 | Methanogenesis, acetate => methane | | | | |
| | | M00358 | Coenzyme M biosynthesis | | | | |
| | | M00378 | F420 biosynthesis | | | | |
| | | M00422 | Acetyl-CoA pathway, CO2 => acetyl-CoA | | | | |
| | | M00563 | Methanogenesis, methylamine/dimethylamine/trimethylamine => ... | | | | |
| | | M00567 | Methanogenesis, CO2 => methane | | | | |
| | | M00608 | 2-Oxocarboxylic acid chain extension, 2-oxoglutarate => 2-oxoadi... | | | | |
| map00710 | Carbon fixation in photosynthetic organisms | M00165 | Reductive pentose phosphate cycle (Calvin cycle) | | | | |
| | | M00166 | Reductive pentose phosphate cycle, ribulose-5P => glyceraldehyd... | | | | |
| | | M00167 | Reductive pentose phosphate cycle, glyceraldehyde-3P => ribulos... | | | | |
| | | M00168 | CAM (Crassulacean acid metabolism), dark | | | | |
| | | M00169 | CAM (Crassulacean acid metabolism), light | | | | |
| | | M00170 | C4-dicarboxylic acid cycle, phosphoenolpyruvate carboxykinase ... | | | | |
| | | M00171 | C4-dicarboxylic acid cycle, NAD - malic enzyme type | | | | |
| | | M00172 | C4-dicarboxylic acid cycle, NADP - malic enzyme type | | | | |
| map00720 | Carbon fixation pathways in prokaryotes | M00173 | Reductive citrate cycle (Arnon-Buchanan cycle) | | | | |
| | | M00374 | Dicarboxylate-hydroxybutyrate cycle | | | | |
| | | M00375 | Hydroxypropionate-hydroxybutylate cycle | | | | |
| | | M00376 | 3-Hydroxypropionate bi-cycle | | | | |
| | | M00377 | Reductive acetyl-CoA pathway (Wood-Ljungdahl pathway) | | | | |
| | | M00579 | Phosphate acetyltransferase-acetate kinase pathway, acetyl-CoA ... | | | | |
| | | M00620 | Incomplete reductive citrate cycle, acetyl-CoA => oxoglutarate | | | | |
| map00730 | Thiamine metabolism | M00127 | Thiamine biosynthesis, AIR => thiamine-P/thiamine-2P | | | | |
| map00740 | Riboflavin metabolism | M00125 | Riboflavin biosynthesis, GTP => riboflavin/FMN/FAD | | | | |
| map00750 | Vitamin B6 metabolism | M00124 | Pyridoxal biosynthesis, erythrose-4P => pyridoxal-5P | | | | |
| map00760 | Nicotinate and nicotinamide metabolism | M00115 | NAD biosynthesis, aspartate => NAD | | | | |
| | | M00622 | Nicotinate degradation, nicotinate => fumarate | | | | |
| map00770 | Pantothenate and CoA biosynthesis | M00119 | Pantothenate biosynthesis, valine/L-aspartate => pantothenate | | | | |
| | | M00120 | Coenzyme A biosynthesis, pantothenate => CoA | | | | |
| map00780 | Biotin metabolism | M00123 | Biotin biosynthesis, pimeloyl-ACP/CoA => biotin | | | | |
| | | M00572 | Pimeloyl-ACP biosynthesis, BioC-BioH pathway, malonyl-ACP => ... | | | | |
| | | M00573 | Biotin biosynthesis, BioI pathway, long-chain-acyl-ACP => pimelo... | | | | |
| | | M00577 | Biotin biosynthesis, BioW pathway, pimelate => pimeloyl-CoA => ... | | | | |
| map00790 | Folate biosynthesis | M00126 | Tetrahydrofolate biosynthesis, GTP => THF | | | | |
| map00860 | Porphyrin and chlorophyll metabolism | M00121 | Heme biosynthesis, glutamate => protoheme/siroheme | | | | |
| | | M00122 | Cobalamin biosynthesis, cobinamide => cobalamin | | | | |
| map00900 | Terpenoid backbone biosynthesis | M00095 | C5 isoprenoid biosynthesis, mevalonate pathway | | | | |
| | | M00096 | C5 isoprenoid biosynthesis, non-mevalonate pathway | | | | |
| | | M00364 | C10-C20 isoprenoid biosynthesis, bacteria | | | | |
| | | M00365 | C10-C20 isoprenoid biosynthesis, archaea | | | | |
| | | M00366 | C10-C20 isoprenoid biosynthesis, plants | | | | |
| | | M00367 | C10-C20 isoprenoid biosynthesis, non-plant eukaryotes | | | | |
| map00905 | Brassinosteroid biosynthesis | M00371 | Castasterone biosynthesis, campesterol => castasterone | | | | |
| map00906 | Carotenoid biosynthesis | M00097 | beta-Carotene biosynthesis, GGAP => beta-carotene | | | | |
| | | M00372 | Abscisic acid biosynthesis, beta-carotene => abscisic acid | | | | |
| map00910 | Nitrogen metabolism | M00175 | Nitrogen fixation, nitrogen => ammonia | | | | |
| | | M00528 | Nitrification, ammonia => nitrite | | | | |
| | | M00529 | Denitrification, nitrate => nitrogen | | | | |
| | | M00530 | Dissimilatory nitrate reduction, nitrate => ammonia | | | | |
| | | M00531 | Assimilatory nitrate reduction, nitrate => ammonia | | | | |
| map00920 | Sulfur metabolism | M00176 | Assimilatory sulfate reduction, sulfate => H2S | | | | |
| | | M00595 | Thiosulfate oxidation by SOX complex, thiosulfate => sulfate | | | | |
| | | M00596 | Dissimilatory sulfate reduction, sulfate => H2S | | | | |
| map00940 | Phenylpropanoid biosynthesis | M00039 | Monolignol biosynthesis, phenylalanine/tyrosine | | | | |
| | | M00137 | Flavanone biosynthesis, phenylalanine => naringenin | | | | |
| map00941 | Flavonoid biosynthesis | M00137 | Flavanone biosynthesis, phenylalanine => naringenin | | | | |
| | | M00138 | Flavonoid biosynthesis, naringenin => pelargonidin | | | | |
| map00966 | Glucosinolate biosynthesis | M00370 | Glucosinolate biosynthesis, tryptophan => glucobrassicin | | | | |
| map00970 | Aminoacyl-tRNA biosynthesis | M00359 | Aminoacyl-tRNA biosynthesis, eukaryotes | | | | |
| | | M00360 | Aminoacyl-tRNA biosynthesis, prokaryotes | | | | |
| map01200 | Carbon metabolism | M00001 | Glycolysis (Embden-Meyerhof pathway), glucose => pyruvate | | | | |
| | | M00002 | Glycolysis, core module involving three-carbon compounds | | | | |
| | | M00004 | Pentose phosphate pathway (Pentose phosphate cycle) | | | | |
| | | M00005 | PRPP biosynthesis, ribose 5P => PRPP | | | | |
| | | M00006 | Pentose phosphate pathway, oxidative phase, glucose 6P => ribul... | | | | |
| | | M00007 | Pentose phosphate pathway, non-oxidative phase, fructose 6P =>... | | | | |
| | | M00008 | Entner-Doudoroff pathway, glucose-6P => glyceraldehyde-3P + p... | | | | |
| | | M00009 | Citrate cycle (TCA cycle, Krebs cycle) | | | | |
| | | M00010 | Citrate cycle, first carbon oxidation, oxaloacetate => 2-oxoglutarate | | | | |
| | | M00011 | Citrate cycle, second carbon oxidation, 2-oxoglutarate => oxaloac... | | | | |
| | | M00012 | Glyoxylate cycle | | | | |
| | | M00013 | Malonate semialdehyde pathway, propanoyl-CoA => Acetyl-CoA | | | | |
| | | M00020 | Serine biosynthesis, glycerate-3P => serine | | | | |
| | | M00021 | Cysteine biosynthesis, serine => cysteine | | | | |
| | | M00165 | Reductive pentose phosphate cycle (Calvin cycle) | | | | |
| | | M00166 | Reductive pentose phosphate cycle, ribulose-5P => glyceraldehyd... | | | | |
| | | M00167 | Reductive pentose phosphate cycle, glyceraldehyde-3P => ribulos... | | | | |
| | | M00168 | CAM (Crassulacean acid metabolism), dark | | | | |
| | | M00169 | CAM (Crassulacean acid metabolism), light | | | | |
| | | M00170 | C4-dicarboxylic acid cycle, phosphoenolpyruvate carboxykinase ... | | | | |
| | | M00171 | C4-dicarboxylic acid cycle, NAD - malic enzyme type | | | | |
| | | M00172 | C4-dicarboxylic acid cycle, NADP - malic enzyme type | | | | |
| | | M00173 | Reductive citrate cycle (Arnon-Buchanan cycle) | | | | |
| | | M00174 | Methane oxidation, methanotroph, methane => formaldehyde | | | | |
| | | M00307 | Pyruvate oxidation, pyruvate => acetyl-CoA | | | | |
| | | M00308 | Semi-phosphorylative Entner-Doudoroff pathway, gluconate => gl... | | | | |
| | | M00309 | Non-phosphorylative Entner-Doudoroff pathway, gluconate/galact... | | | | |
| | | M00344 | Formaldehyde assimilation, xylulose monophosphate pathway | | | | |
| | | M00345 | Formaldehyde assimilation, ribulose monophosphate pathway | | | | |
| | | M00346 | Formaldehyde assimilation, serine pathway | | | | |
| | | M00356 | Methanogenesis, methanol => methane | | | | |
| | | M00357 | Methanogenesis, acetate => methane | | | | |
| | | M00374 | Dicarboxylate-hydroxybutyrate cycle | | | | |
| | | M00375 | Hydroxypropionate-hydroxybutylate cycle | | | | |
| | | M00376 | 3-Hydroxypropionate bi-cycle | | | | |
| | | M00377 | Reductive acetyl-CoA pathway (Wood-Ljungdahl pathway) | | | | |
| | | M00563 | Methanogenesis, methylamine/dimethylamine/trimethylamine => ... | | | | |
| | | M00567 | Methanogenesis, CO2 => methane | | | | |
| | | M00579 | Phosphate acetyltransferase-acetate kinase pathway, acetyl-CoA ... | | | | |
| | | M00580 | Pentose phosphate pathway, archaea, fructose 6P => ribose 5P | | | | |
| | | M00620 | Incomplete reductive citrate cycle, acetyl-CoA => oxoglutarate | | | | |
| | | M00633 | Semi-phosphorylative Entner-Doudoroff pathway, gluconate/galac... | | | | |
| map01210 | 2-Oxocarboxylic acid metabolism | M00010 | Citrate cycle, first carbon oxidation, oxaloacetate => 2-oxoglutarate | | | | |
| | | M00019 | Valine/isoleucine biosynthesis, pyruvate => valine / 2-oxobutanoat... | | | | |
| | | M00028 | Ornithine biosynthesis, glutamate => ornithine | | | | |
| | | M00031 | Lysine biosynthesis, 2-aminoadipate => lysine | | | | |
| | | M00033 | Ectoine biosynthesis, aspartate => ectoine | | | | |
| | | M00370 | Glucosinolate biosynthesis, tryptophan => glucobrassicin | | | | |
| | | M00432 | Leucine biosynthesis, 2-oxoisovalerate => 2-oxoisocaproate | | | | |
| | | M00433 | Lysine biosynthesis, 2-oxoglutarate => 2-oxoadipate | | | | |
| | | M00535 | Isoleucine biosynthesis, pyruvate => 2-oxobutanoate | | | | |
| | | M00608 | 2-Oxocarboxylic acid chain extension, 2-oxoglutarate => 2-oxoadi... | | | | |
| map01212 | Fatty acid metabolism | M00082 | Fatty acid biosynthesis, initiation | | | | |
| | | M00083 | Fatty acid biosynthesis, elongation | | | | |
| | | M00085 | Fatty acid biosynthesis, elongation, mitochondria | | | | |
| | | M00086 | beta-Oxidation, acyl-CoA synthesis | | | | |
| | | M00087 | beta-Oxidation | | | | |
| | | M00415 | Fatty acid biosynthesis, elongation, endoplasmic reticulum | | | | |
| map01220 | Degradation of aromatic compounds | M00174 | Methane oxidation, methanotroph, methane => formaldehyde | | | | |
| | | M00418 | Toluene degradation, anaerobic, toluene => benzoyl-CoA | | | | |
| | | M00419 | Cymene degradation, p-cymene => p-cumate | | | | |
| | | M00533 | Homoprotocatechuate degradation, homoprotocatechuate => 2-ox... | | | | |
| | | M00534 | Naphthalene degradation, naphthalene => salicylate | | | | |
| | | M00537 | Xylene degradation, xylene => methylbenzoate | | | | |
| | | M00538 | Toluene degradation, toluene => benzoate | | | | |
| | | M00539 | Cumate degradation, p-cumate => 2-oxopent-4-enoate + 2-methyl... | | | | |
| | | M00540 | Benzoate degradation, cyclohexanecarboxylic acid =>pimeloyl-CoA | | | | |
| | | M00541 | Benzoyl-CoA degradation, benzoyl-CoA => 3-hydroxypimeloyl-CoA | | | | |
| | | M00543 | Biphenyl degradation, biphenyl => 2-oxopent-4-enoate + benzoate | | | | |
| | | M00544 | Carbazole degradation, carbazole => 2-oxopent-4-enoate + anthr... | | | | |
| | | M00545 | Trans-cinnamate degradation, trans-cinnamate => acetyl-CoA | | | | |
| | | M00547 | Benzene/toluene degradation, benzene => catechol / toluene => 3... | | | | |
| | | M00548 | Benzene degradation, benzene => catechol | | | | |
| | | M00551 | Benzoate degradation, benzoate => catechol / methylbenzoate =>... | | | | |
| | | M00568 | Catechol ortho-cleavage, catechol => 3-oxoadipate | | | | |
| | | M00569 | Catechol meta-cleavage, catechol => acetyl-CoA / 4-methylcatech... | | | | |
| | | M00623 | Phthalate degradation, phthalate => protocatechuate | | | | |
| | | M00624 | Terephthalate degradation, terephthalate => 3,4-dihydroxybenzoate | | | | |
| | | M00636 | Phthalate degradation, phthalate => protocatechuate | | | | |
| map01230 | Biosynthesis of amino acids | M00002 | Glycolysis, core module involving three-carbon compounds | | | | |
| | | M00005 | PRPP biosynthesis, ribose 5P => PRPP | | | | |
| | | M00007 | Pentose phosphate pathway, non-oxidative phase, fructose 6P =>... | | | | |
| | | M00015 | Proline biosynthesis, glutamate => proline | | | | |
| | | M00016 | Lysine biosynthesis, succinyl-DAP pathway, aspartate => lysine | | | | |
| | | M00017 | Methionine biosynthesis, apartate => homoserine => methionine | | | | |
| | | M00018 | Threonine biosynthesis, aspartate => homoserine => threonine | | | | |
| | | M00019 | Valine/isoleucine biosynthesis, pyruvate => valine / 2-oxobutanoat... | | | | |
| | | M00020 | Serine biosynthesis, glycerate-3P => serine | | | | |
| | | M00021 | Cysteine biosynthesis, serine => cysteine | | | | |
| | | M00022 | Shikimate pathway, phosphoenolpyruvate + erythrose-4P => chori... | | | | |
| | | M00023 | Tryptophan biosynthesis, chorismate => tryptophan | | | | |
| | | M00024 | Phenylalanine biosynthesis, chorismate => phenylalanine | | | | |
| | | M00025 | Tyrosine biosynthesis, chorismate => tyrosine | | | | |
| | | M00026 | Histidine biosynthesis, PRPP => histidine | | | | |
| | | M00028 | Ornithine biosynthesis, glutamate => ornithine | | | | |
| | | M00029 | Urea cycle | | | | |
| | | M00030 | Lysine biosynthesis, AAA pathway, 2-oxoglutarate => 2-aminoadi... | | | | |
| | | M00031 | Lysine biosynthesis, 2-aminoadipate => lysine | | | | |
| | | M00033 | Ectoine biosynthesis, aspartate => ectoine | | | | |
| | | M00040 | Tyrosine biosynthesis, prephanate => pretyrosine => tyrosine | | | | |
| | | M00338 | Cysteine biosynthesis, homocysteine + serine => cysteine | | | | |
| | | M00432 | Leucine biosynthesis, 2-oxoisovalerate => 2-oxoisocaproate | | | | |
| | | M00433 | Lysine biosynthesis, 2-oxoglutarate => 2-oxoadipate | | | | |
| | | M00525 | Lysine biosynthesis, acetyl-DAP pathway, aspartate => lysine | | | | |
| | | M00526 | Lysine biosynthesis, DAP dehydrogenase pathway, aspartate => l... | | | | |
| | | M00527 | Lysine biosynthesis, DAP aminotransferase pathway, aspartate =... | | | | |
| | | M00535 | Isoleucine biosynthesis, pyruvate => 2-oxobutanoate | | | | |
| | | M00570 | Isoleucine biosynthesis, threonine => 2-oxobutanoate => isoleucine | | | | |
| | | M00580 | Pentose phosphate pathway, archaea, fructose 6P => ribose 5P | | | | |
| | | M00609 | Cysteine biosynthesis, methionine => cysteine | | | | |
| map01501 | beta-Lactam resistance | M00625 | Methicillin resistance | | | | |
| | | M00627 | beta-Lactam resistance, Bla system | | | | |
| | | M00628 | beta-Lactam resistance, AmpC system | | | | |
| | | M00649 | Multidrug resistance, efflux pump AdeABC | | | | |
| | | M00654 | ParS-ParR (polymyxin-adaptive resistance) two-component regul... | | | | |
| | | M00655 | AdeS-AdeR two-component regulatory system | | | | |
| map02010 | ABC transporters | M00185 | Sulfate transport system | | | | |
| | | M00186 | Tungstate transport system | | | | |
| | | M00189 | Molybdate transport system | | | | |
| | | M00190 | Iron(III) transport system | | | | |
| | | M00191 | Thiamine transport system | | | | |
| | | M00194 | Maltose/maltodextrin transport system | | | | |
| | | M00196 | Raffinose/stachyose/melibiose transport system | | | | |
| | | M00198 | Putative sn-glycerol-phosphate transport system | | | | |
| | | M00199 | L-Arabinose/lactose transport system | | | | |
| | | M00200 | Putative sorbitol/mannitol transport system | | | | |
| | | M00201 | alpha-Glucoside transport system | | | | |
| | | M00202 | Oligogalacturonide transport system | | | | |
| | | M00203 | Glucose/arabinose transport system | | | | |
| | | M00204 | Trehalose/maltose transport system | | | | |
| | | M00205 | N-Acetylglucosamine transport system | | | | |
| | | M00206 | Cellobiose transport system | | | | |
| | | M00208 | Glycine betaine/proline transport system | | | | |
| | | M00209 | Osmoprotectant transport system | | | | |
| | | M00210 | Phospholipid transport system | | | | |
| | | M00212 | Ribose transport system | | | | |
| | | M00213 | L-Arabinose transport system | | | | |
| | | M00214 | Methyl-galactoside transport system | | | | |
| | | M00215 | D-Xylose transport system | | | | |
| | | M00216 | Multiple sugar transport system | | | | |
| | | M00217 | D-Allose transport system | | | | |
| | | M00218 | Fructose transport system | | | | |
| | | M00219 | AI-2 transport system | | | | |
| | | M00220 | Rhamnose transport system | | | | |
| | | M00222 | Phosphate transport system | | | | |
| | | M00223 | Phosphonate transport system | | | | |
| | | M00224 | Fluoroquinolones transport system | | | | |
| | | M00225 | Lysine/arginine/ornithine transport system | | | | |
| | | M00226 | Histidine transport system | | | | |
| | | M00227 | Glutamine transport system | | | | |
| | | M00228 | Putative glutamine transport system | | | | |
| | | M00229 | Arginine transport system | | | | |
| | | M00230 | Glutamate/aspartate transport system | | | | |
| | | M00231 | Octopine/nopaline transport system | | | | |
| | | M00232 | General L-amino acid transport system | | | | |
| | | M00233 | Glutamate transport system | | | | |
| | | M00234 | Cystine transport system | | | | |
| | | M00235 | Arginine/ornithine transport system | | | | |
| | | M00237 | Branched-chain amino acid transport system | | | | |
| | | M00238 | D-Methionine transport system | | | | |
| | | M00239 | Peptides/nickel transport system | | | | |
| | | M00240 | Iron complex transport system | | | | |
| | | M00241 | Vitamin B12 transport system | | | | |
| | | M00242 | Zinc transport system | | | | |
| | | M00245 | Cobalt/nickel transport system | | | | |
| | | M00246 | Nickel transport system | | | | |
| | | M00249 | Capsular polysaccharide transport system | | | | |
| | | M00250 | Lipopolysaccharide transport system | | | | |
| | | M00251 | Teichoic acid transport system | | | | |
| | | M00252 | Lipooligosaccharide transport system | | | | |
| | | M00253 | Sodium transport system | | | | |
| | | M00255 | Lipoprotein-releasing system | | | | |
| | | M00256 | Cell division transport system | | | | |
| | | M00257 | Hemin transport system | | | | |
| | | M00259 | Heme transport system | | | | |
| | | M00298 | Multidrug/hemolysin transport system | | | | |
| | | M00299 | Spermidine/putrescine transport system | | | | |
| | | M00300 | Putrescine transport system | | | | |
| | | M00301 | Mannopine transport system | | | | |
| | | M00302 | 2-Aminoethylphosphonate transport system | | | | |
| | | M00314 | Bacitracin transport system | | | | |
| | | M00316 | Manganese transport system | | | | |
| | | M00317 | Manganese/iron transport system | | | | |
| | | M00318 | Iron/zinc/copper transport system | | | | |
| | | M00319 | Manganese/zinc/iron transport system | | | | |
| | | M00320 | Lipopolysaccharide export system | | | | |
| | | M00321 | Bicarbonate transport system | | | | |
| | | M00322 | Neutral amino acid transport system | | | | |
| | | M00323 | Urea transport system | | | | |
| | | M00324 | Dipeptide transport system | | | | |
| | | M00325 | alpha-Hemolysin/cyclolysin transport system | | | | |
| | | M00348 | Glutathione transport system | | | | |
| | | M00349 | Microcin C transport system | | | | |
| | | M00423 | Molybdate/tungstate transport system | | | | |
| | | M00435 | Taurine transport system | | | | |
| | | M00436 | Sulfonate transport system | | | | |
| | | M00437 | Phthalate transport system | | | | |
| | | M00438 | Nitrate/nitrite transport system | | | | |
| | | M00439 | Oligopeptide transport system | | | | |
| | | M00440 | Nickel transport system | | | | |
| | | M00442 | Putative hydroxymethylpyrimidine transport system | | | | |
| | | M00491 | arabinogalactan oligomer/maltooligosaccharide transport system | | | | |
| | | M00566 | Dipeptide transport system, Firmicutes | | | | |
| | | M00581 | Biotin transport system | | | | |
| | | M00582 | Energy-coupling factor transport system | | | | |
| | | M00583 | Putative peptide transport system | | | | |
| | | M00584 | Acetoin utilization transport system | | | | |
| | | M00585 | L-Cystine transport system | | | | |
| | | M00586 | Putative amino-acid transport system | | | | |
| | | M00587 | Arginine/lysine/histidine/glutamine transport system | | | | |
| | | M00589 | Putative lysine transport system | | | | |
| | | M00590 | Erythritol transport system | | | | |
| | | M00591 | Putative xylitol transport system | | | | |
| | | M00592 | Inositol transport system | | | | |
| | | M00593 | Inositol transport system | | | | |
| | | M00599 | Inositol-phosphate transport system | | | | |
| | | M00600 | alpha-1,4-Digalacturonate transport system | | | | |
| | | M00601 | Putative chitobiose transport system | | | | |
| | | M00602 | Arabinosaccharide transport system | | | | |
| | | M00603 | Putative aldouronate transport system | | | | |
| | | M00604 | Trehalose transport system | | | | |
| | | M00605 | Glucose/mannose transport system | | | | |
| | | M00606 | N,N'-Diacetylchitobiose transport system | | | | |
| | | M00607 | Glycerol transport system | | | | |
| | | M00619 | Xylobiose transport system | | | | |
| | | M00634 | Oleandomycin transport system | | | | |
| | | M00635 | Tetracycline resistance, TetAB(46) transporter | | | | |
| map02020 | Two-component system | M00314 | Bacitracin transport system | | | | |
| | | M00315 | Uncharacterized ABC transport system | | | | |
| | | M00316 | Manganese transport system | | | | |
| | | M00434 | PhoR-PhoB (phosphate starvation response) two-component regu... | | | | |
| | | M00443 | SenX3-RegX3 (phosphate starvation response) two-component re... | | | | |
| | | M00444 | PhoQ-PhoP (magnesium transport) two-component regulatory sy... | | | | |
| | | M00445 | EnvZ-OmpR (osmotic stress response) two-component regulatory... | | | | |
| | | M00446 | RstB-RstA two-component regulatory system | | | | |
| | | M00447 | CpxA-CpxR (envelope stress response) two-component regulator... | | | | |
| | | M00448 | CssS-CssR (secretion stress response) two-component regulator... | | | | |
| | | M00449 | CreC-CreB (phosphate regulation) two-component regulatory sy... | | | | |
| | | M00450 | BaeS-BaeR (envelope stress response) two-component regulator... | | | | |
| | | M00451 | BasS-BasR (antimicrobial peptide resistance) two-component reg... | | | | |
| | | M00452 | CusS-CusR (copper tolerance) two-component regulatory system | | | | |
| | | M00453 | QseC-QseB (quorum sensing) two-component regulatory system | | | | |
| | | M00454 | KdpD-KdpE (potassium transport) two-component regulatory sy... | | | | |
| | | M00455 | TorS-TorR (TMAO respiration) two-component regulatory system | | | | |
| | | M00456 | ArcB-ArcA (anoxic redox control) two-component regulatory system | | | | |
| | | M00457 | TctE-TctD (tricarboxylic acid transport) two-component regulatory ... | | | | |
| | | M00458 | ResE-ResD (aerobic and anaerobic respiration) two-component r... | | | | |
| | | M00459 | VicK-VicR (cell wall metabolism) two-component regulatory system | | | | |
| | | M00460 | MprB-MprA (maintenance of persistent infection) two-component r... | | | | |
| | | M00461 | MtrB-MtrA (osmotic stress response) two-component regulatory s... | | | | |
| | | M00462 | PrrB-PrrA (intracellular multiplication) two-component regulatory s... | | | | |
| | | M00463 | TrcS-TrcR two-component regulatory system | | | | |
| | | M00464 | NrsS-NrsR (nickel tolerance) two-component regulatory system | | | | |
| | | M00465 | ManS-ManR (manganese homeostasis) two-component regulator... | | | | |
| | | M00466 | NblS-NblR (photosynthesis) two-component regulatory system | | | | |
| | | M00467 | SasA-RpaAB (circadian timing mediating) two-component regulat... | | | | |
| | | M00468 | SaeS-SaeR (staphylococcal virulence regulation) two-component ... | | | | |
| | | M00469 | BceS-BceR (bacitracin transport) two-component regulatory system | | | | |
| | | M00470 | YxdK-YxdJ (antimicrobial peptide response) two-component regul... | | | | |
| | | M00471 | NarX-NarL (nitrate respiration) two-component regulatory system | | | | |
| | | M00472 | NarQ-NarP (nitrate respiration) two-component regulatory system | | | | |
| | | M00473 | UhpB-UhpA (hexose phosphates uptake) two-component regulato... | | | | |
| | | M00474 | RcsC-RcsD-RcsB (capsule synthesis) two-component regulatory ... | | | | |
| | | M00475 | BarA-UvrY (central carbon metabolism) two-component regulatory... | | | | |
| | | M00476 | ComP-ComA (competence) two-component regulatory system | | | | |
| | | M00477 | EvgS-EvgA (acid and drug tolerance) two-component regulatory s... | | | | |
| | | M00478 | DegS-DegU (multicellular behavior control) two-component regula... | | | | |
| | | M00479 | DesK-DesR (membrane lipid fluidity regulation) two-component re... | | | | |
| | | M00480 | VraS-VraR (cell-wall peptidoglycan synthesis) two-component reg... | | | | |
| | | M00481 | LiaS-LiaR (cell wall stress response) two-component regulatory sy... | | | | |
| | | M00482 | DevS-DevR (redox response) two-component regulatory system | | | | |
| | | M00483 | NreB-NreC (dissimilatory nitrate/nitrite reduction) two-component r... | | | | |
| | | M00484 | YdfH-YdfI two-component regulatory system | | | | |
| | | M00485 | KinABCDE-Spo0FA (sporulation control) two-component regulator... | | | | |
| | | M00486 | CitA-CitB (citrate fermentation) two-component regulatory system | | | | |
| | | M00487 | CitS-CitT (magnesium-citrate transport) two-component regulatory... | | | | |
| | | M00488 | DcuS-DcuR (C4-dicarboxylate metabolism) two-component regulat... | | | | |
| | | M00489 | DctS-DctR (C4-dicarboxylate transport) two-component regulatory... | | | | |
| | | M00490 | MalK-MalR (malate transport) two-component regulatory system | | | | |
| | | M00492 | LytS-LytR two-component regulatory system | | | | |
| | | M00493 | AlgZ-AlgR (alginate production) two-component regulatory system | | | | |
| | | M00494 | NatK-NatR (sodium extrusion) two-component regulatory system | | | | |
| | | M00495 | AgrC-AgrA (exoprotein synthesis) two-component regulatory sy... | | | | |
| | | M00496 | ComD-ComE (competence) two-component regulatory system | | | | |
| | | M00497 | GlnL-GlnG (nitrogen regulation) two-component regulatory system | | | | |
| | | M00498 | NtrY-NtrX (nitrogen regulation) two-component regulatory system | | | | |
| | | M00499 | HydH-HydG (metal tolerance) two-component regulatory system | | | | |
| | | M00500 | AtoS-AtoC (cPHB biosynthesis) two-component regulatory system | | | | |
| | | M00501 | PilS-PilR (type 4 fimbriae synthesis) two-component regulatory sy... | | | | |
| | | M00502 | GlrK-GlrR (amino sugar metabolism) two-component regulatory s... | | | | |
| | | M00503 | PgtB-PgtA (phosphoglycerate transport) two-component regulator... | | | | |
| | | M00504 | DctB-DctD (C4-dicarboxylate transport) two-component regulatory... | | | | |
| | | M00505 | KinB-AlgB (alginate production) two-component regulatory system | | | | |
| | | M00506 | CheA-CheYBV (chemotaxis) two-component regulatory system | | | | |
| | | M00507 | ChpA-ChpB/PilGH (chemosensory) two-component regulatory sy... | | | | |
| | | M00508 | PixL-PixGH (positive phototaxis) two-component regulatory system | | | | |
| | | M00509 | WspE-WspRF (chemosensory) two-component regulatory system | | | | |
| | | M00510 | Cph1-Rcp1 (light response) two-component regulatory system | | | | |
| | | M00511 | PleC-PleD (cell fate control) two-component regulatory system | | | | |
| | | M00512 | CckA-CtrA/CpdR (cell cycle control) two-component regulatory sy... | | | | |
| | | M00513 | LuxQN/CqsS-LuxU-LuxO (quorum sensing) two-component regul... | | | | |
| | | M00514 | TtrS-TtrR (tetrathionate respiration) two-component regulatory sy... | | | | |
| | | M00515 | FlrB-FlrC (polar flagellar synthesis) two-component regulatory sy... | | | | |
| | | M00516 | SLN1-YPD1-SSK1/SKN7 (osmosensing) two-component regulato... | | | | |
| | | M00517 | RpfC-RpfG (cell-to-cell signaling) two-component regulatory system | | | | |
| | | M00518 | GlnK-GlnL (glutamine utilization) two-component regulatory system | | | | |
| | | M00519 | YesM-YesN two-component regulatory system | | | | |
| | | M00520 | ChvG-ChvI (acidity sensing) two-component regulatory system | | | | |
| | | M00521 | CiaH-CiaR two-component regulatory system | | | | |
| | | M00522 | SalK-SalR two-component regulatory system | | | | |
| | | M00523 | RegB-RegA (redox response) two-component regulatory system | | | | |
| | | M00524 | FixL-FixJ (nitrogen fixation) two-component regulatory system | | | | |
| | | M00645 | Multidrug resistance, efflux pump SmeABC | | | | |
| | | M00646 | Multidrug resistance, efflux pump AcrAD-TolC | | | | |
| | | M00648 | Multidrug resistance, efflux pump MdtABC | | | | |
| | | M00651 | Vancomycin resistance, D-Ala-D-Lac type | | | | |
| | | M00652 | Vancomycin resistance, D-Ala-D-Ser type | | | | |
| | | M00653 | AauS-AauR (acidic amino acids utilization) two-component regula... | | | | |
| | | M00654 | ParS-ParR (polymyxin-adaptive resistance) two-component regul... | | | | |
| | | M00656 | VanS-VanR (VanB type vancomycin resistance) two-component r... | | | | |
| | | M00657 | VanS-VanR (VanE type vancomycin resistance) two-component r... | | | | |
| | | M00658 | VanS-VanR (actinomycete type vancomycin resistance) two-com... | | | | |
| | | M00662 | Hk1-Rrp1 (glycerol uptake and utilization) two-component regulat... | | | | |
| | | M00663 | SsrA-SsrB two-component regulatory system | | | | |
| | | M00716 | ArlS-ArlR (virulence regulation) two-component regulatory system | | | | |
| map02030 | Bacterial chemotaxis | M00506 | CheA-CheYBV (chemotaxis) two-component regulatory system | | | | |
| map02060 | Phosphotransferase system (PTS) | M00265 | PTS system, glucose-specific II component | | | | |
| | | M00267 | PTS system, N-acetylglucosamine-specific II component | | | | |
| | | M00268 | PTS system, arbutin-like II component | | | | |
| | | M00269 | PTS system, sucrose-specific II component | | | | |
| | | M00270 | PTS system, trehalose-specific II component | | | | |
| | | M00271 | PTS system, beta-glucosides-specific II component | | | | |
| | | M00272 | PTS system, arbutin-, cellobiose-, and salicin-specific II component | | | | |
| | | M00273 | PTS system, fructose-specific II component | | | | |
| | | M00274 | PTS system, mannitol-specific II component | | | | |
| | | M00275 | PTS system, cellobiose-specific II component | | | | |
| | | M00276 | PTS system, mannose-specific II component | | | | |
| | | M00277 | PTS system, N-acetylgalactosamine-specific II component | | | | |
| | | M00278 | PTS system, sorbose-specific II component | | | | |
| | | M00279 | PTS system, galactitol-specific II component | | | | |
| | | M00280 | PTS system, glucitol/sorbitol-specific II component | | | | |
| | | M00281 | PTS system, lactose-specific II component | | | | |
| | | M00282 | PTS system, D-glucosamine-specific II component | | | | |
| | | M00283 | PTS system, ascorbate-specific II component | | | | |
| | | M00287 | PTS system, galactosamine-specific II component | | | | |
| | | M00303 | PTS system, N-acetylmuramic acid-specific II component | | | | |
| | | M00304 | PTS system, fructose-specific II component | | | | |
| | | M00305 | PTS system, 2-O-A-mannosyl-D-glycerate-specific II component | | | | |
| | | M00610 | PTS system, D-glucosaminate-specific II component | | | | |
| map03008 | Ribosome biogenesis in eukaryotes | M00425 | H/ACA ribonucleoprotein complex | | | | |
| map03010 | Ribosome | M00177 | Ribosome, eukaryotes | | | | |
| | | M00178 | Ribosome, bacteria | | | | |
| | | M00179 | Ribosome, archaea | | | | |
| map03013 | RNA transport | M00399 | Cap binding complex | | | | |
| | | M00405 | THC complex | | | | |
| | | M00406 | TREX complex | | | | |
| | | M00426 | Survival motor neuron (SMN) complex | | | | |
| | | M00427 | Nuclear pore complex | | | | |
| | | M00428 | eIF4F complex | | | | |
| | | M00430 | Exon junction complex (EJC) | | | | |
| map03015 | mRNA surveillance pathway | M00399 | Cap binding complex | | | | |
| | | M00430 | Exon junction complex (EJC) | | | | |
| map03018 | RNA degradation | M00390 | Exosome, archaea | | | | |
| | | M00391 | Exosome, eukaryotes | | | | |
| | | M00392 | Ski complex | | | | |
| | | M00393 | TRAMP complex | | | | |
| | | M00394 | RNA degradosome | | | | |
| | | M00395 | Decapping complex | | | | |
| | | M00396 | Lsm 2-8 complex | | | | |
| | | M00397 | Lsm 1-7 complex | | | | |
| map03020 | RNA polymerase | M00180 | RNA polymerase II, eukaryotes | | | | |
| | | M00181 | RNA polymerase III, eukaryotes | | | | |
| | | M00182 | RNA polymerase I, eukaryotes | | | | |
| | | M00183 | RNA polymerase, bacteria | | | | |
| | | M00184 | RNA polymerase, archaea | | | | |
| map03030 | DNA replication | M00260 | DNA polymerase III complex, bacteria | | | | |
| | | M00261 | DNA polymerase alpha / primase complex | | | | |
| | | M00262 | DNA polymerase delta complex | | | | |
| | | M00263 | DNA polymerase epsilon complex | | | | |
| | | M00285 | MCM complex | | | | |
| | | M00288 | RPA complex | | | | |
| | | M00289 | RF-C complex | | | | |
| map03040 | Spliceosome | M00351 | Spliceosome, U1-snRNP | | | | |
| | | M00352 | Spliceosome, U2-snRNP | | | | |
| | | M00353 | Spliceosome, Prp19/CDC5L complex | | | | |
| | | M00354 | Spliceosome, U4/U6.U5 tri-snRNP | | | | |
| | | M00355 | Spliceosome, 35S U5-snRNP | | | | |
| | | M00396 | Lsm 2-8 complex | | | | |
| | | M00398 | Sm core complex | | | | |
| | | M00405 | THC complex | | | | |
| | | M00406 | TREX complex | | | | |
| map03050 | Proteasome | M00337 | Immunoproteasome | | | | |
| | | M00340 | Proteasome, 20S core particle | | | | |
| | | M00341 | Proteasome, 19S regulatory particle (PA700) | | | | |
| | | M00342 | Bacterial proteasome | | | | |
| | | M00343 | Archaeal proteasome | | | | |
| map03060 | Protein export | M00401 | Sec61 complex | | | | |
| map03070 | Bacterial secretion system | M00325 | alpha-Hemolysin/cyclolysin transport system | | | | |
| | | M00331 | Type II general secretion pathway | | | | |
| | | M00332 | Type III secretion system | | | | |
| | | M00333 | Type IV secretion system | | | | |
| | | M00334 | Type VI secretion system | | | | |
| | | M00335 | Sec (secretion) system | | | | |
| | | M00336 | Twin-arginine translocation (Tat) system | | | | |
| map03410 | Base excision repair | M00262 | DNA polymerase delta complex | | | | |
| | | M00296 | BER complex | | | | |
| map03420 | Nucleotide excision repair | M00288 | RPA complex | | | | |
| | | M00289 | RF-C complex | | | | |
| | | M00290 | Holo-TFIIH complex | | | | |
| | | M00385 | Cul4-DDB1-DDB2 complex | | | | |
| | | M00386 | Cul4-DDB1-CSA complex | | | | |
| map03430 | Mismatch repair | M00260 | DNA polymerase III complex, bacteria | | | | |
| | | M00295 | BRCA1-associated genome surveillance complex (BASC) | | | | |
| map03440 | Homologous recombination | M00291 | MRN complex | | | | |
| | | M00292 | MRX complex | | | | |
| | | M00295 | BRCA1-associated genome surveillance complex (BASC) | | | | |
| map03450 | Non-homologous end-joining | M00292 | MRX complex | | | | |
| | | M00297 | DNA-PK complex | | | | |
| map03460 | Fanconi anemia pathway | M00293 | DNA polymerase zeta | | | | |
| | | M00413 | FA core complex | | | | |
| | | M00414 | Bloom's syndrome complex | | | | |
| map04010 | MAPK signaling pathway | M00687 | MAPK (ERK1/2) signaling | | | | |
| | | M00688 | MAPK (JNK) signaling | | | | |
| | | M00689 | MAPK (p38) signaling | | | | |
| | | M00690 | MAPK (ERK5) signaling | | | | |
| map04011 | MAPK signaling pathway - yeast | M00516 | SLN1-YPD1-SSK1/SKN7 (osmosensing) two-component regulato... | | | | |
| map04013 | MAPK signaling pathway - fly | M00687 | MAPK (ERK1/2) signaling | | | | |
| | | M00688 | MAPK (JNK) signaling | | | | |
| | | M00689 | MAPK (p38) signaling | | | | |
| map04022 | cGMP-PKG signaling pathway | M00694 | cGMP signaling | | | | |
| map04024 | cAMP signaling pathway | M00695 | cAMP signaling | | | | |
| map04064 | NF-kappa B signaling pathway | M00686 | Toll-like receptor signaling | | | | |
| map04068 | FoxO signaling pathway | M00676 | PI3K-Akt signaling | | | | |
| map04110 | Cell cycle | M00284 | Origin recognition complex | | | | |
| | | M00285 | MCM complex | | | | |
| | | M00297 | DNA-PK complex | | | | |
| | | M00381 | SCF-SKP2 complex | | | | |
| | | M00389 | APC/C complex | | | | |
| | | M00691 | DNA damage-induced cell cycle checkpoints | | | | |
| | | M00692 | Cell cycle - G1/S transition | | | | |
| | | M00693 | Cell cycle - G2/M transition | | | | |
| map04111 | Cell cycle-yeast | M00284 | Origin recognition complex | | | | |
| | | M00379 | SCF-MET30 complex | | | | |
| | | M00407 | SCF-CDC4 complex | | | | |
| | | M00411 | SCF-GRR1 complex | | | | |
| | | M00692 | Cell cycle - G1/S transition | | | | |
| | | M00693 | Cell cycle - G2/M transition | | | | |
| map04112 | Cell cycle - Caulobacter | M00511 | PleC-PleD (cell fate control) two-component regulatory system | | | | |
| | | M00512 | CckA-CtrA/CpdR (cell cycle control) two-component regulatory sy... | | | | |
| map04114 | Oocyte meiosis | M00380 | SCF-BTRC complex | | | | |
| map04120 | Ubiquitin mediated proteolysis | M00379 | SCF-MET30 complex | | | | |
| | | M00380 | SCF-BTRC complex | | | | |
| | | M00381 | SCF-SKP2 complex | | | | |
| | | M00382 | SCF-FBS complex | | | | |
| | | M00383 | ECV complex | | | | |
| | | M00384 | Cul3-SPOP complex | | | | |
| | | M00385 | Cul4-DDB1-DDB2 complex | | | | |
| | | M00386 | Cul4-DDB1-CSA complex | | | | |
| | | M00387 | SCF-FBW7 complex | | | | |
| | | M00388 | ECS complex | | | | |
| | | M00389 | APC/C complex | | | | |
| | | M00407 | SCF-CDC4 complex | | | | |
| | | M00411 | SCF-GRR1 complex | | | | |
| map04141 | Protein processing in endoplasmic reticulum | M00382 | SCF-FBS complex | | | | |
| | | M00400 | p97-Ufd1-Npl4 complex | | | | |
| | | M00401 | Sec61 complex | | | | |
| | | M00402 | Translocon-associated protein (TRAP) complex | | | | |
| | | M00403 | HRD1/SEL1 ERAD complex | | | | |
| | | M00404 | COPII complex | | | | |
| map04144 | Endocytosis | M00408 | ESCRT-0 complex | | | | |
| | | M00409 | ESCRT-I complex | | | | |
| | | M00410 | ESCRT-II complex | | | | |
| | | M00412 | ESCRT-III complex | | | | |
| map04151 | PI3K-Akt signaling pathway | M00676 | PI3K-Akt signaling | | | | |
| map04210 | Apoptosis | M00685 | Apoptotic machinery | | | | |
| map04310 | Wnt signaling pathway | M00677 | Wnt signaling | | | | |
| map04330 | Notch signaling pathway | M00682 | Notch signaling | | | | |
| map04340 | Hedgehog signaling pathway | M00678 | Hedgehog signaling | | | | |
| map04350 | TGF-beta signaling pathway | M00679 | BMP signaling | | | | |
| | | M00680 | TGF-beta signaling | | | | |
| | | M00681 | Activin signaling | | | | |
| map04390 | Hippo signaling pathway | M00679 | BMP signaling | | | | |
| | | M00680 | TGF-beta signaling | | | | |
| | | M00683 | Hippo signaling | | | | |
| map04391 | Hippo signaling pathway - fly | M00683 | Hippo signaling | | | | |
| map04550 | Signaling pathways regulating pluripotency ... | M00681 | Activin signaling | | | | |
| map04620 | Toll-like receptor signaling pathway | M00686 | Toll-like receptor signaling | | | | |
| map04623 | Cytosolic DNA-sensing pathway | M00181 | RNA polymerase III, eukaryotes | | | | |
| map04626 | Plant-pathogen interaction | M00339 | RaxAB-RaxC type I secretion system | | | | |
| map04630 | Jak-STAT signaling pathway | M00684 | JAK-STAT signaling | | | | |
| map05016 | Huntington's disease | M00180 | RNA polymerase II, eukaryotes | | | | |
| map05111 | Vibrio cholerae pathogenic cycle | M00513 | LuxQN/CqsS-LuxU-LuxO (quorum sensing) two-component regul... | | | | |
| | | M00515 | FlrB-FlrC (polar flagellar synthesis) two-component regulatory sy... | | | | |
| map05120 | Epithelial cell signaling in Helicobacter pylo... | M00564 | Helicobacter pylori pathogenicity signature, cagA pathogenicity is... | | | | |
| map05130 | Pathogenic Escherichia coli infection | M00363 | EHEC pathogenicity signature, Shiga toxin | | | | |
| | | M00542 | EHEC/EPEC pathogenicity signature, T3SS and effectors | | | | |
| map05131 | Shigellosis | M00363 | EHEC pathogenicity signature, Shiga toxin | | | | |
| map05133 | Pertussis | M00574 | Pertussis pathogenicity signature 1, pertussis toxin | | | | |
| | | M00575 | Pertussis pathogenicity signature 2, T1SS | | | | |
| map05211 | Renal cell carcinoma | M00383 | ECV complex | | | | |
| map05217 | Basal cell carcinoma | M00678 | Hedgehog signaling | | | | |
