# Supplementary material for: Assessing biosynthetic potential of agricultural groundwater through metagenomic sequencing: A diverse anammox community dominates nitrate-rich groundwater
Source: PLoS One. 2017 Apr 6;12(4):e0174930. doi: 10.1371/journal.pone.0174930 (PMC5383146; doi:10.1371/journal.pone.0174930)
Supplement: S11 Table — (DOCX) [file pone.0174930.s016.docx]

Table S11 – Number FASTQ sequence reads passing QC

|  |  |  | **singletons** | |
| --- | --- | --- | --- | --- |
| **Sample** | **merged** | **paired** | **R1** | **R2** |
| Domestic | 17,905,782 | 35,042,733 | 1,687,107 | 157,722 |
| Lagoon | 12,041,749 | 51,202,883 | 1,465,521 | 224,644 |
| mw5 | 6,248,020 | 29,671,197 | 1,225,811 | 129,184 |
| mw6 | 6,149,998 | 38,965,159 | 1,884,433 | 162,203 |
