# Supplementary material for: Assessing biosynthetic potential of agricultural groundwater through metagenomic sequencing: A diverse anammox community dominates nitrate-rich groundwater
Source: PLoS One. 2017 Apr 6;12(4):e0174930. doi: 10.1371/journal.pone.0174930 (PMC5383146; doi:10.1371/journal.pone.0174930)
Supplement: S12 Table — (DOCX) [file pone.0174930.s017.docx]

Table S12 – Total bp for FASTQ sequences for reads passing QC

|  |  | **paired** | | **singletons** | |
| --- | --- | --- | --- | --- | --- |
| **sample** | **merged** | **R1** | **R2** | **R1** | **R2** |
| domestic | 2,230,231,777 | 3,475,116,427 | 3,334,096,468 | 149,919,937 | 13,527,864 |
| lagoon | 1,457,197,301 | 5,065,107,077 | 4,936,494,896 | 128,588,226 | 19,623,461 |
| mw5 | 739,422,547 | 2,941,567,504 | 2,832,399,048 | 108,237,531 | 11,223,151 |
| mw6 | 756,756,489 | 3,870,539,861 | 3,700,505,595 | 167,467,895 | 14,117,759 |
