# Supplementary material for: Assessing biosynthetic potential of agricultural groundwater through metagenomic sequencing: A diverse anammox community dominates nitrate-rich groundwater
Source: PLoS One. 2017 Apr 6;12(4):e0174930. doi: 10.1371/journal.pone.0174930 (PMC5383146; doi:10.1371/journal.pone.0174930)
Supplement: S16 Table — (DOCX) [file pone.0174930.s021.docx]

Table S16 – Bioinformatics software used

| **Name** | **Version/Date** | **Usage** |
| --- | --- | --- |
| Price [1] | 1.2 04/08/2014 | Reference-guided assembly |
| AntiSMASH 3 [2-5] | 20a8957 06/12/2015 | Biosynthetic gene cluster identification |
| PrinSeq-lite [6] | 0.20.4 11/10/2013 | Quality filtering FASTQ reads |
| Prokka [8] | f06d6de 11/12/2014 | ORF finding and functional annotation of contigs |
| BLAST+ [9-15][[more](http://blast.ncbi.nlm.nih.gov/blast/Blast.cgi?CMD=Web&PAGE_TYPE=BlastDocs&DOC_TYPE=References)] | 2.2.31 6/15/2015 | search query sequences against database |
| RAPSearch 2 [16, 17] | 2.22 10/27/2014 | search query sequences against database |
| Bowtie 2 [18, 19] | 2.2.2 04/10/2014 | align query sequences to reference |
| REAPR [20] | 1.0.17 11/24/2014 | Locating positions to break contigs |
| HMMER 2 [21] | 2.3.2 10/04/2003 | AntiSMASH |
| HMMER 3 [22] | 3.1b1 05/26/2013 | AntiSMASH and single-copy genes |
| SAMtools [23, 24][[more](http://www.htslib.org/doc/)] | 1.2 02/03/2015 | analyze Bowtie2 outputs |
| MetaPhlAn [27] | 2f1b17a 06/13/2014 | Relative abundances using clade-specific markers |
| MetaPhlAn 2 [27] | 901cc57 06/25/2015 | Relative abundances using clade-specific markers |
| Arb {Ludwig, 2004 #4288} | 6.0.6 08/22/2016 | 16S phylogeny |
| IDBA-UD {Peng, 2012 #3000} | 10/31/2012 | metagenome assembly |
| BRIG {Alikhan, 2011 #4287} | 07/10/2012 | graphic comparison of microbial genomes |
| CheckM {Parks, 2015 #3982} | 1.0.6 04/23/2016 | check quality of metagenomics bins |
| VizBin {Laczny, 2015 #3676} | 01/20/2015 | bin genomes from metagenome assembly |
| EMIRGE {Miller, 2011 #1816} | b3ab71c 07/03/2012 | assemble 16S RNA genes from shotgun sequence |
| USEARCH {Edgar, 2015 #4286} | 8 09/29/2014 | merge paired end reads |

**Representative UNIX shell code for launching bioinformatics software:**

**PRICE TARGETTED ASSEMBLY:**

PriceTI -a 14 -fpp MW-6_Oct2012_R1_filt33.fastq MW-6_Oct2012_R2_filt33.fastq 500 97 -icf mw6.vizbin2_09_rpkmFilt_1.fa 2 2 4 -icfNt mw6.vizbin2_09_16S_price.cycle3.fa 2 2 4 -nc 1 -target 95 0 -lenf 500 0 -lenf 1000 1 -o mw6.vizbin2_09_16S_price3.fa -logf mw6.vizbin2_09_16S_price3.log

**PRINSEQ-LITE READ FILTERING:**

#Shell loop through all the R1 and R2 files:

for i in ${R1[@]}; do nohup prinseq-lite -fastq ${R1[$count]} -fastq2 ${R2[$count]} -no_qual_header -min_len 85 -min_gc 10 -max_gc 90 -min_qual_mean 30 -ns_max_n 1 -lc_method entropy -lc_threshold 5 -trim_qual_left 30 -trim_qual_right 30 -trim_qual_type min -trim_qual_rule lt -trim_qual_window 1 -trim_qual_step 1 2> trim.prinseq.err & ((count++)); done

**USEARCH8 MERGE READ PAIRS:**

#merge read pairs in parallel

declare -a R1=(*_R1_prinseq_good_*.fastq) #get names

declare -a R2=(*_R2_prinseq_good_*.fastq)

count=0 # now run a loop

for i in ${R1[@]}; do nohup usearch8 -fastq_mergepairs ${R1[$count]} -reverse ${R2[$count]} -fastqout ${R1[$count]:0:-27}.merged.fastq -fastqout_notmerged_fwd ${R1[$count]:0:-27}.notmerged.fastq -fastqout_notmerged_rev ${R2[$count]:0:-27}.notmerged.fastq -label_suffix " merged" -fastq_allowmergestagger -fastq_trimstagger -fastq_qmax 93 -fastq_qmaxout 93 & ((count++)); done

**ANTISMASH BIOSYNTHETIC GENE CLUSTER FINDING:**

while read -r p; do mkdir antismash_${p:0:-4}; run_antismash.py --verbose --cpus 15 --logfile antismash_${p:0:-4}/antismash_${p:0:-4}.log --statusfile antismash_${p:0:-4}/antismash_${p:0:-4}.status --input-type nucl --clusterblast --subclusterblast --smcogs --knownclusterblast --inclusive --borderpredict --nclusters 100 ${p} --outputfolder antismash_${p:0:-4} > antismash_${p:0:-4}/antismash_${p:0:-4}.out 2> antismash_${p:0:-4}/antismash_${p:0:-4}.err; done <names.txt&> antismashLoop.err &

**PROKKA GENOME ANNOTATION:**

while read -r p; do prokka --outdir prokka_${p:0:-3} --cpus 4 --rnammer --locustag ${p:0:-3} ${p} > prokka_${p:0:-3}.out 2> prokka_${p:0:-3}.err; done <names.txt &> prokka_while.out &

**IDBA-UD ASSEMBLY:**

./fq2fa --merge --filter dairy-domestic.r1.prinseq.fastq dairy-domestic.r2.prinseq.fastq dairy-dom.prinseq.merged.fa

./idba_ud -r dairy-dom.prinseq.merged.fa --pre_correction -o idba-ud_assembly_v1 &

**CHECKM METAGENOMIC BIN QC:**

# run checkM using its own databases

checkm lineage_wf -x fna -t 12 --pplacer_threads 12 -f checkm_lineage.out -l 0.4 -u 5 --tab_table . checkm_v1 &> checkm_v1.out &

# note that ‘-l 0.4’ is used due to the low sequence homology of these genomes to available databases

#plot results as bar graph:

checkm bin_qa_plot -x fa checkm_v1/ . checkm_v1/plots &

#run checkm with Mads Albertsen’s 111 single copy genes hmm:

checkm analyze -t 24 -x fa ~/essentialgenes.hmm . checkm_v1/ &

checkm qa -t 24 ~/essentialgenes.hmm checkm_v1/ &

**EMIRGE ASSEMBLE 16S rRNA GENES FROM SHOTGUN METAGENOME:**

python emirge.py dom/ -1 dom_R1.fastq -2 dom_R2.fastq -f dom_db.fix.fasta -b dom_db -l 101 -i 350 -s 150 -a 20 -v 0.1 -j 0.99 --phred33 & > dom.out &

References

1. Ruby JG, Bellare P, DeRisi JL. PRICE: Software for the Targeted Assembly of Components of (Meta) Genomic Sequence Data. G3: Genes|Genomes|Genetics. 2013;3(5):865-80. doi: 10.1534/g3.113.005967.

2. Medema MH, Blin K, Cimermancic P, de Jager V, Zakrzewski P, Fischbach MA, et al. antiSMASH: rapid identification, annotation and analysis of secondary metabolite biosynthesis gene clusters in bacterial and fungal genome sequences. Nucleic Acids Research. 2011;39(suppl 2):W339-W46. doi: 10.1093/nar/gkr466.

3. Weber T, Blin K, Duddela S, Krug D, Kim HU, Bruccoleri R, et al. antiSMASH 3.0—a comprehensive resource for the genome mining of biosynthetic gene clusters. Nucleic Acids Research. 2015. doi: 10.1093/nar/gkv437.

4. Blin K, Medema MH, Kazempour D, Fischbach MA, Breitling R, Takano E, et al. antiSMASH 2.0—a versatile platform for genome mining of secondary metabolite producers. Nucleic Acids Research. 2013;41(W1):W204-W12. doi: 10.1093/nar/gkt449.

5. Cimermancic P, Medema Marnix H, Claesen J, Kurita K, Wieland Brown Laura C, Mavrommatis K, et al. Insights into Secondary Metabolism from a Global Analysis of Prokaryotic Biosynthetic Gene Clusters. Cell. 2014;158(2):412-21. doi: http://dx.doi.org/10.1016/j.cell.2014.06.034.

6. Schmieder R, Edwards R. Quality control and preprocessing of metagenomic datasets. Bioinformatics. 2011;27(6):863-4. doi: 10.1093/bioinformatics/btr026.

7. Martin M. Cutadapt removes adapter sequences from high-throughput sequencing reads. 2011. 2011;17(1). doi: 10.14806/ej.17.1.200

pp. 10-12.

8. Seemann T. Prokka: rapid prokaryotic genome annotation. Bioinformatics. 2014;30(14):2068-9. doi: 10.1093/bioinformatics/btu153.

9. Altschul SF, Gish W, Miller W, Myers EW, Lipman DJ. Basic local alignment search tool. Journal of Molecular Biology. 1990;215(3):403-10. doi: http://dx.doi.org/10.1016/S0022-2836(05)80360-2.

10. Gish W, States DJ. Identification of protein coding regions by database similarity search. Nat Genet. 1993;3(3):266-72.

11. Altschul SF, Madden TL, Schäffer AA, Zhang J, Zhang Z, Miller W, et al. Gapped BLAST and PSI-BLAST: a new generation of protein database search programs. Nucleic Acids Research. 1997;25(17):3389-402. doi: 10.1093/nar/25.17.3389.

12. Karlin S, Altschul SF. Methods for assessing the statistical significance of molecular sequence features by using general scoring schemes. Proceedings of the National Academy of Sciences. 1990;87(6):2264-8.

13. Karlin S, Altschul SF. Applications and statistics for multiple high-scoring segments in molecular sequences. Proceedings of the National Academy of Sciences. 1993;90(12):5873-7.

14. Zhang Z, Miller W, Schäffer AA, Madden TL, Lipman DJ, Koonin EV, et al. Protein sequence similarity searches using patterns as seeds. Nucleic Acids Research. 1998;26(17):3986-90. doi: 10.1093/nar/26.17.3986.

15. Camacho C, Coulouris G, Avagyan V, Ma N, Papadopoulos J, Bealer K, et al. BLAST+: architecture and applications. BMC Bioinformatics. 2009;10(1):421. PubMed PMID: doi:10.1186/1471-2105-10-421.

16. Ye Y, Choi J-H, Tang H. RAPSearch: a fast protein similarity search tool for short reads. BMC Bioinformatics. 2011;12(1):159. PubMed PMID: doi:10.1186/1471-2105-12-159.

17. Zhao Y, Tang H, Ye Y. RAPSearch2: a fast and memory-efficient protein similarity search tool for next-generation sequencing data. Bioinformatics. 2012;28(1):125-6. doi: 10.1093/bioinformatics/btr595.

18. Langmead B, Salzberg SL. Fast gapped-read alignment with Bowtie 2. Nat Meth. 2012;9(4):357-9. doi: 10.1038/nmeth.1923

http://www.nature.com/nmeth/journal/v9/n4/abs/nmeth.1923.html#supplementary-information.

19. Langmead B, Trapnell C, Pop M, Salzberg S. Ultrafast and memory-efficient alignment of short DNA sequences to the human genome. Genome Biology. 2009;10(3):R25. PubMed PMID: doi:10.1186/gb-2009-10-3-r25.

20. Hunt M, Kikuchi T, Sanders M, Newbold C, Berriman M, Otto T. REAPR: a universal tool for genome assembly evaluation. Genome Biology. 2013;14(5):R47. PubMed PMID: doi:10.1186/gb-2013-14-5-r47.

21. Howard Hughes Medical Institute, Washington University School of Medicine. HMMER 2 2003. Available from: http://hmmer.org.

22. Howard Hughes Medical Institute. HMMER 3 2013. Available from: http://hmmer.org.

23. Li H, Handsaker B, Wysoker A, Fennell T, Ruan J, Homer N, et al. The Sequence Alignment/Map format and SAMtools. Bioinformatics. 2009;25(16):2078-9. doi: 10.1093/bioinformatics/btp352.

24. Li H. A statistical framework for SNP calling, mutation discovery, association mapping and population genetical parameter estimation from sequencing data. Bioinformatics. 2011;27(21):2987-93. doi: 10.1093/bioinformatics/btr509.

25. Simpson JT, Wong K, Jackman SD, Schein JE, Jones SJM, Birol İ. ABySS: A parallel assembler for short read sequence data. Genome Research. 2009;19(6):1117-23. doi: 10.1101/gr.089532.108.

26. Alneberg J, Bjarnason BS, de Bruijn I, Schirmer M, Quick J, Ijaz UZ, et al. Binning metagenomic contigs by coverage and composition. Nat Meth. 2014;11(11):1144-6. doi: 10.1038/nmeth.3103

http://www.nature.com/nmeth/journal/v11/n11/abs/nmeth.3103.html#supplementary-information.

27. Segata N, Waldron L, Ballarini A, Narasimhan V, Jousson O, Huttenhower C. Metagenomic microbial community profiling using unique clade-specific marker genes. Nat Meth. 2012;9(8):811-4. doi: http://www.nature.com/nmeth/journal/v9/n8/abs/nmeth.2066.html#supplementary-information.

28. Broad Institute. Picard Tools 2009. Available from: http://broadinstitute.github.io/picard/.

29. Darling ACE, Mau B, Blattner FR, Perna NT. Mauve: Multiple Alignment of Conserved Genomic Sequence With Rearrangements. Genome Research. 2004;14(7):1394-403. doi: 10.1101/gr.2289704.

30. Darling AE, Mau B, Perna NT. progressiveMauve: Multiple Genome Alignment with Gene Gain, Loss and Rearrangement. PLoS ONE. 2010;5(6):e11147. doi: 10.1371/journal.pone.0011147.

31. Rissman AI, Mau B, Biehl BS, Darling AE, Glasner JD, Perna NT. Reordering contigs of draft genomes using the Mauve Aligner. Bioinformatics. 2009;25(16):2071-3. doi: 10.1093/bioinformatics/btp356.
